# Stemness-associated MEF-derived extruded nanovesicles cooperate with *Lactobacillus rhamnosus* to alleviate DSS-induced colitis through mucosal microenvironment remodeling

**DOI:** 10.1039/d6na00298f

**Published:** 2026-07-21

**Authors:** Xinyu Cao, Chao Yan, Qing Ji, Yutong Pan, Wencheng Shi, Huijun Zhao, Aihua Gong, Zhengzou Fang

**Affiliations:** a Department of Laboratory Medicine, Affiliated Hospital of Jiangsu University Zhenjiang 212001 China myfangzz@163.com; b The Affiliated Huai'an Hospital of Xuzhou Medical University and The Second People's Hospital of Huai'an No. 62, Huaihai Road (S) Huai'an 223002 China

## Abstract

*Lactobacillus rhamnosus* (*L. rhamnosus*) can modulate intestinal microbiota, decrease harmful bacterial metabolites, and thereby improve the intestinal microenvironment of patients with ulcerative colitis (UC). However, rapid inactivation and low colonization efficiency caused by intestinal peristalsis and impaired mucosa severely restrict its therapeutic outcomes. Extracellular vesicles (EVs) exhibit excellent mucus-penetrating ability that enables them to reach deep intestinal crypts. Subsequently, EVs directly deliver repair signals to intestinal epithelial cells and immune cells, effectively promoting intestinal mucosal repair. In this study, we prepared stemness-associated mouse embryonic fibroblast-derived extruded nanovesicles (sMEF-eNVs) *via* small-molecule intervention and three-dimensional (3D) culture. The prepared sMEF-eNVs displayed nanoscale morphology, EV-associated phenotype expression, and physicochemical features consistent with those of EVs. In a DSS-induced mouse model of UC, sMEF-eNVs improved epithelial barrier integrity, increased tight-junction and mucus-associated barrier signals, and attenuated mucosal inflammatory responses. Combined administration of sMEF-eNVs and *L. rhamnosus* further alleviated disease activity, improved histological injury, modulated Th17/Treg-associated immune imbalance, and was accompanied by shifts in gut microbial composition and fecal metabolic profiles. These findings support a vesicle-probiotic combination strategy for intestinal inflammation.

## Abstract 2

Combined sMEF-eNVs and *L. rhamnosus* treatment improved epithelial barrier integrity and Th17/Treg balance, offering a multimodal strategy for alleviating DSS-induced colitis (image created with Biorender).

## Graphical abstract

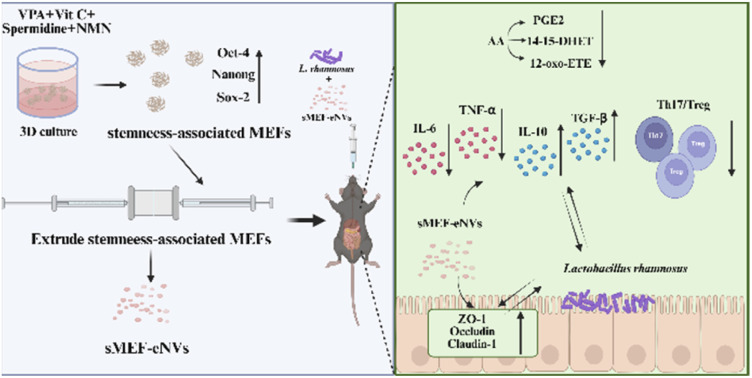

## 1 Introduction

Ulcerative colitis (UC) is a chronic relapsing inflammatory bowel disease characterized by epithelial barrier disruption, gut microbiota dysbiosis, and mucosal immune imbalance.^1,2^ In genetically susceptible hosts, environmental stimuli and microbial antigens can trigger excessive immune activation, resulting in increased intestinal permeability, inflammatory cell infiltration, and persistent mucosal injury.^1,3^ Imbalance between pro-inflammatory and regulatory T-cell responses, particularly Th17/Treg disequilibrium, further contributes to cytokine network dysregulation and amplifies the barrier-microbiota-immune inflammatory loop.^4^

Probiotic interventions in patients with UC can significantly improve gut microbiota diversity and elevate the abundance of beneficial bacteria and the levels of short-chain fatty acids (SCFAs), thereby effectively restoring intestinal microecological balance.^5^*L. rhamnosus* can competitively inhibit pathogens, promote mucin secretion, upregulate tight-junction proteins, and produce SCFAs, making it a promising probiotic candidate for UC intervention.^6,7^ However, during the acute or chronic active phases of UC, a severely damaged mucosal barrier, a highly inflammatory local microenvironment, reduced adhesion sites, and significant alterations in mucus-layer composition collectively impair the adhesion and functional activity of exogenous probiotics in the intestinal mucosa.^6,8^ Therefore, strategies that improve the mucosal microenvironment may enhance probiotic-associated therapeutic effects.^9^

Extracellular vesicles (EVs) are nanoscale membrane-bound vesicles that can carry bioactive vesicular components, including proteins, lipids, and nucleic acids, from donor cells.^9,10^ Previous studies have implicated signaling pathways such as Wnt/beta-catenin, STAT3, TGF-beta/Smad, PI3K/Akt, and NF-κB-related pathways in EV-mediated epithelial repair and immunomodulation.^11–15^ However, the use of natural EVs is constrained by critical bottlenecks, including low production yields, substantial batch-to-batch variability, and high costs associated with large-scale manufacturing.^10,16^

Given the bottleneck in yield and standardized production of natural EVs, mimetic technologies for preparing cell-derived nanovesicles (CNVs) *via* mechanical or membrane extrusion have emerged.^16,17^ The yield of CNVs can be increased by one to two orders of magnitude compared to natural EVs, and the therapeutic efficacy of CNVs can be comparable to that of natural EVs.^17–19^ However, the biological efficacy of EVs should be evaluated not only in terms of yield but also in terms of functional potency, with the physiological state of donor cells serving as a key determinant of vesicle potency.^10,16^ To obtain high-functional-potency CNVs, a 3D spheroid culture system and chemical reprogramming and small-molecule intervention were used to maintain and enhance the stemness-associated markers of MEFs.^20–23^ In this study, valproic acid (VPA), vitamin C, spermidine, and nicotinamide mononucleotide (NMN) were selected as candidate small molecules because they target complementary epigenetic and metabolic pathways involved in cellular plasticity and stemness. VPA, a histone deacetylase inhibitor, enhances histone acetylation and chromatin accessibility, thereby promoting stemness-associated transcription and improving somatic cell reprogramming efficiency.^24^ Vitamin C facilitates epigenetic remodeling by acting as a cofactor for ten-eleven translocation (TET) and Jumonji C domain-containing histone demethylase (JHDM) family demethylases, leading to the activation of endogenous stemness-associated genes during reprogramming.^25,26^ Spermidine was included because polyamine metabolism supports stem-cell self-renewal, while spermidine supplementation helps maintain proliferative and differentiation potential by reducing oxidative stress and delaying cellular senescence through SIRT3-dependent mechanisms.^27,28^ NMN, a direct NAD^+^ precursor, restores NAD^+^ homeostasis and enhances mitochondrial function, energy production, and antioxidant capacity *via* the NAD^+^/SIRT3 pathway, thereby supporting cellular fitness and stemness-related properties.^29^

**Scheme 1:**
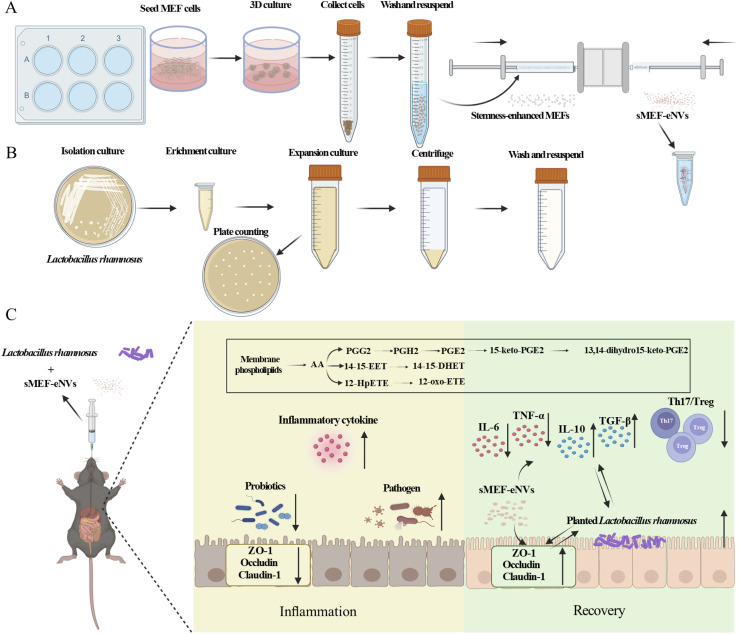
Schematic illustration of the preparation of *L. rhamnosus* and sMEF-eNVs and their synergistic therapeutic mechanism in UC. (A) Demonstration of a synthetic procedure of sMEF-eNVs. (B) The cultivation process of *L. rhamnosus*. (C) The mechanism of sMEF-eNVs combined with *L. rhamnosus* for UC synergistic therapy *in vivo*. Created with Biorender.com.

Herein, we prepared extruded nanovesicles derived from MEFs with enhanced stemness-associated marker expression and further verified their synergistic therapeutic effects with *L. rhamnosus* in a DSS-induced UC mouse model. First, we activated and enhanced the stemness characteristics of MEFs through small molecule intervention combined with 3D culture technology. Subsequently, sMEF-eNVs were produced from these stemness-associated MEFs by membrane extrusion technology (Scheme 1A). Then, sMEF-eNVs combined with *L. rhamnosus* was used to treat mice with DSS-induced UC (Scheme 1B). During treatment, sMEF-eNVs upregulated the expression of tight junction proteins and mucins, thereby repairing the damaged intestinal barrier. Simultaneously, sMEF-eNVs restored the Th17/Treg balance, alleviated mucosal inflammation, and re-established immune homeostasis, improving the inflammatory and barrier-disrupted intestinal microenvironment and potentially supporting the functional activity of *L. rhamnosus*. *L. rhamnosus* restores intestinal microbial diversity, strengthens the intestinal mucosal barrier and immune homeostasis, and thereby sustains intestinal microecological balance (Scheme 1C). These findings provide a theoretical and experimental foundation for developing novel “vesicle-probiotic” combined biotherapeutic strategies.

## 2 Methods and materials

### 2.1 Drug solution preparation

Valproic acid (VPA, S161023, Aladdin, Shanghai, China, purity ≥ 98%), vitamin C (VitC, A5960, Merck KGaA, Darmstadt, Germany, purity ≥ 99%), β-nicotinamide mononucleotide (NMN, HY-F0004, MedChemExpress, New Jersey, USA, purity = 99.94%), and spermidine (S107071, Aladdin, Shanghai, China, purity ≥ 99%) were individually dissolved in PBS to prepare 100 mmol L^−1^ stock solutions. These were filter-sterilized using a 0.22-µm filter in a biosafety cabinet and stored at −20 °C.^30–32^ During the experiment, they were diluted to working concentrations using DMEM.

### 2.2 3D MEF cell culture

First, 1 g of agarose was dissolved in 100 mL distilled water and autoclaved to obtain a 1% agarose solution. 25 mL of the above solution was mixed with 25 mL of pre-warmed Dulbecco's Modified Eagle Medium (DMEM, MA0212, MeilunBio, Liaoning, China), and three milliliters of the mixture was added to each well of a six-well plate, and the plate was dried in a biosafety cabinet for 2 h.^33,34^ The dried plates could be stored at 4 °C for no longer than one week. Subsequently, MEF cells from 2D culture were collected and counted. The cell suspension was added dropwise to the agarose-coated plates at a density of 12 000 cells per well.^35^ The plate was gently rocked several times to ensure a single-cell suspension and then incubated for 5 days.^36^ After culture, the medium was transferred to a new sterile 15 mL centrifuge tube using a pipette and centrifuged at 1200 rpm for 3 min. The supernatant was discarded, and the cell pellet was washed with PBS and finally collected into a 1.5 mL microcentrifuge tube. All MEF cell culture experiments were performed using three independent cell-culture experiments unless otherwise specified. When technical replicates were included within an experiment, they were averaged to generate one value for each independent biological experiment.

### 2.3 Preparation and purification of sMEF-eNVs

First, MEF cells subjected to small-molecule and 3D culture conditioning were collected, washed twice with pre-cooled PBS, and resuspended in pre-cooled PBS at approximately 1 × 10^7^ cells per mL. Using a liposome extruder (Hand Extruder-1 mL, Genizer LLC, California, USA), the suspension was sequentially extruded through polycarbonate membranes with pore sizes of 1 µm (610010, Avanti, Alabama, USA), 0.8 µm (610009, Avanti, Alabama, USA), 0.4 µm (610007, Avanti, Alabama, USA), and 0.2 µm (610006, Avanti, Alabama, USA).^37^ Approximately 10 extrusions were performed for each pore size to obtain a homogeneous nanovesicle suspension.^38^ Samples, buffers, and collection tubes were maintained under low-temperature conditions whenever possible to minimize protein degradation and vesicle aggregation. Subsequently, the extruded sample underwent differential centrifugation at 4 °C: sequentially at 300 g for 10 min, 2000 g for 20 min, and 10 000 g for 30 min to remove unbroken cells, nuclei, and debris.^39^ The supernatant was collected, and vesicles were pelleted by ultracentrifugation (Optima XPN-90, Beckman, California, USA). The pellet was re-suspended in PBS and washed by centrifugation at 100 000 g for 1 h to improve purity.^39^ Finally, purified vesicles were re-suspended in sterile PBS, aliquoted, and stored at −80 °C, avoiding repeated freeze–thaw cycles.^39,40^ For characterization, a 10-µL aliquot of the sMEF-eNVs suspension was placed on a copper grid, allowed to settle for 1 min, stained with 10 µL phosphotungstic acid for 1 min, dried at room temperature, and observed under TEM at 80–120 kV.^40^ Besides, nanoparticle tracking analysis was used to determine particle concentration and size distribution, and a zeta potential analyzer was used to detect surface potential. Subsequent experiments used doses standardized based on the particle number.

### 2.4 Animal experiments

This study strictly followed the Jiangsu University Guidelines for the Care and Use of Laboratory Animals. All animal protocols were approved by the Jiangsu University Animal Ethics Committee (UJS-IACUC-AP-2022030272). Thirty healthy specific-pathogen-free (SPF) male C57BL/6J mice (6 weeks old) were purchased from Changzhou Cavens Experimental Animal Co., Ltd.^41^ Mice were housed in SPF animal facilities at Jiangsu University Medical School under controlled temperature and humidity conditions and a 12 h light/12 h dark cycle, with free access to food and water.^42^ Mice were housed in groups of five per individually ventilated cage (IVC) with autoclaved corncob bedding and a red translucent plastic shelter for environmental enrichment. After one week of acclimatization, mice were randomly divided into 6 groups (*n* = 5/group) using a random number table. Researchers blinded to group assignments assessed body weight, fecal consistency, and bleeding daily during the experiment. To minimize distress, mice were provided with softened chow on the cage floor from day 4 of DSS administration onward, ensuring easy access to food. No analgesics or anti-inflammatory drugs were administered, as these could have confounded the colitis-related endpoints. Humane endpoints were predefined as any of the following: weight loss exceeding 30% of initial body weight, or severe bloody diarrhea accompanied by inactivity. Mice reaching these endpoints were immediately euthanized using an isoflurane overdose followed by cervical dislocation, and the same method was used at the scheduled termination. Throughout the experiment, no animals met the criteria for early euthanasia and no unexpected adverse events occurred.

The UC model was established using DSS. Except for the normal control group, mice in the other groups received 2.5% DSS in sterile drinking water *ad libitum* from day 0 to day 7, followed by normal drinking water until sample collection on day 14. Treatments were administered once daily by oral gavage from day 0 to day 14.^42^ The DAI score was calculated daily based on weight loss, fecal consistency, and fecal blood, with specific scoring criteria shown in Table 1 (SI).^42,43^ The intervention protocols were as follows: the normal control and DSS model groups received daily oral gavage of PBS (100 µL per mouse per day), the *L. rhamnosus* group received daily oral gavage of *L. rhamnosus* (3 × 10^9^ CFU per mouse per day, 100 µL), the MEF-eNVs group received daily oral gavage of MEF-eNVs (1 × 10^10^ particles per mouse per day, 100 µL), the sMEF-eNVs group received daily oral gavage of sMEF-eNVs (1 × 10^10^ particles per mouse per day, 100 µL), and the combined therapy group received daily oral gavage of both *L. rhamnosus* (3 × 10^9^ CFU per mouse per day, 100 µL) and sMEF-eNVs (1 × 10^10^ particles per mouse per day, 100 µL).^41,44^ At the end of the experiment, mice were anesthetized with isoflurane and euthanized. Colon length was measured, and samples were collected. One portion of the colon tissue was fixed in 4% paraformaldehyde for histological analysis, another portion was snap-frozen in liquid nitrogen for molecular assays like qPCR, and fecal samples were stored at −80 °C for subsequent gut microbiota analysis.^45^

**Table 1:** Evaluation criteria of DAI^a^

Score	0	1	2	3	4
Percent body weight loss	0	1–5	5–10	10–20	>20
Stool consistency	Normal	—	Mushy	—	Diarrhea
Rectal bleeding	Negative	—	Positive	—	Visible rectal bleeding

a DAI = (percent body weight loss + stool consistency + rectal bleeding)/3.

### 2.5 Flow cytometry

After euthanasia, mesenteric lymph nodes (MLNs) were immediately excised from each mouse and processed individually, gently homogenized using a syringe plunger, and filtered through a 70 µm cell strainer.^46^ The homogenate was centrifuged at 4 °C, 1000 rpm for 5 min, followed by red blood cell lysis, resuspension, and blocking.^47^ After centrifugation at 4 °C, 1000 rpm for 5 min, cells were resuspended in PBS. The following antibodies and reagents were used for flow cytometry analysis: Zombie Violet™ Fixable Viability Kit (1:500, BioLegend, 423113, California, USA), PE-CD3 (0.5 µg/10^6^ cells, Thermo Fisher Scientific, AB_2815096, MA, USA), APC-CD4 (0.2 µg/10^6^ cells, Thermo Fisher Scientific, AB_2534398, MA, USA), FITC-IL-17 (0.25 µg/10^6^ cells, Thermo Fisher Scientific, AB_763581, MA, USA), and APC-FOXP3 (1 µg/10^6^ cells, Thermo Fisher Scientific, AB_469457, MA, USA). Cells were incubated at 4 °C in the dark for 30 min for surface staining.^47^ To detect Tregs and Th17 cells, intracellular staining was performed using an eBioscience™ Foxp3/transcription factor staining buffer set (Thermo Fisher Scientific, 00-5523-00, MA, USA). After thorough washing and centrifugation, cells were resuspended for flow cytometry analysis.

### 2.6 Histological analysis

Colon tissues were fixed in 4% paraformaldehyde for 24 h, dehydrated through a graded ethanol series, cleared, and embedded in paraffin. Sections 4 and 5 µm thick were cut from the distal colon.^48^ After deparaffinization in xylene and rehydration through graded ethanol, sections were stained.

Hematoxylin and eosin (H&E) staining: deparaffinized and rehydrated sections were immersed in hematoxylin for 5 min, differentiated briefly in acid differentiation solution and blued in ammonia water, rinsed in running water, dehydrated in ethanol, and then immersed in eosin for 2–3 min.^49^ This staining was used to assess mucosal structural integrity, crypt damage, and inflammatory cell infiltration.^50^

Alcian blue staining: deparaffinized and rehydrated sections were first treated with Alcian blue acidification solution and then stained in Alcian blue staining solution (BestBio, BB-44293, Shanghai, China). After rinsing in running water, sections were counterstained with fast red solution, rinsed again, dehydrated, cleared, and mounted with neutral resin.^51^ Digital imaging was performed using a whole-slide scanning system.

### 2.7 RNA extraction and qRT-PCR

Total RNA was extracted from cells using the RNAiso Plus reagent.^52^ Subsequently, RNA was reverse transcribed into cDNA using a reverse transcription kit. Using cDNA as a template, real-time fluorescent quantitative PCR (qRT-PCR) analysis was performed using ChamQ Universal SYBR qPCR Master Mix.^52^ GAPDH was used as the internal reference gene. Relative mRNA expression levels of target genes were calculated using the 2^^−ΔΔ*C*_t_^ method.^53^ The primer sequences used are listed in Table 2 (SI).^43,54^

**Table 2:** Primer sequence for qRT-PCR

Gene	Primers
Oct-4	Forward sequence: 5′-AGAGGATCACCTTGGGGTACA-3′
	Reverse sequence: 5′-CGAAGCGACAGATGGTGGTC-3′
Nanog	Forward sequence: 5′-CACAGTTTGCCTAGTTCTGAGG-3′
	Reverse sequence: 5′-GCAAGAATAGTTCTCGGGATGAA-3′
SOX-2	Forward sequence: 5′-GCGGAGTGGAAACTTTTGTCC-3′
	Reverse sequence: 5′-GGGAAGCGTGTACTTATCCTTCT-3′
GAPDH	Forward sequence: 5′-AGGTCGGTGTGAACGGATTTG-3′
	Reverse sequence: 5′-GGGGTCGTTGATGGCAACA-3′
Claudin1	Forward sequence: 5′-GGGGACAACATCGTGACCG-3′
	Reverse sequence: 5′-AGGAGTCGAAGACTTTGCACT-3′
ZO-1	Forward sequence: 5′-GCTTTAGCGAACAGAAGGAGC-3′
	Reverse sequence: 5′-TTCATTTTTCCGAGACTTCACCA-3′
Occludin	Forward sequence: 5′-TTGAAAGTCCACCTCCTTACAGA-3′
	Reverse sequence: 5′-CCGGATAAAAAGAGTACGCTGG-3′
IL-6	Forward sequence: 5′-TAGTCCTTCCTACCCCAATTTCC-3′
	Reverse sequence: 5′-TTGGTCCTTAGCCACTCCTTC-3′
TNF-α	Forward sequence: 5′-CAGGCGGTGCCTATGTCTC-3′
	Reverse sequence: 5′-CGATCACCCCGAAGTTCAGTAG-3
IL-10	Forward sequence: 5′-GCTCTTACTGACTGGCATGAG-3′
	Reverse sequence: 5′-CGCAGCTCTAGGAGCATGTG-3′
TGF-β	Forward sequence: 5′-CTCCCGTGGCTTCTAGTGC-3′
	Reverse sequence: 5′-GCCTTAGTTTGGACAGGATCTG-3′
16S rRNA	Forward sequence: 5′-ACTCCTACGGGAGGCAGCA-3′
	Reverse sequence: 5′-GGACTACHVGGGTWTCTAAT-3′

### 2.8 Western blot analysis

Cell or tissue samples were lysed on ice for 30 min using RIPA lysis buffer (PS0013, Leagene, Beijing, China) containing protease and phosphatase inhibitors, with periodic vortexing.^55^ After centrifugation at 4 °C, 12 000 rpm for 15 min, the supernatant was collected as total protein extract.^55^ Protein concentration was determined using a bicinchoninic acid assay (BCA) protein detection kit (E112-02, Vazyme, Nanjing, China). Equal amounts of protein samples were mixed with 5× sodium lauryl sulfate (SDS) loading buffer and denatured at 95 °C for 5 min.^55^ After separation by SDS-PAGE, proteins were transferred onto PVDF membranes.^56^ Membranes were blocked at room temperature for 1 h with TBST (Tris-Buffered Saline with Tween 20) containing 5% skim milk.^55^ The primary antibodies were as follows: Oct-4 (1:1000, bs-3816R, Bioss, Beijing, China), Nanog (1:1000, bs-10414R, Bioss, Beijing, China), SOX-2 (1:1000, bs-0523R, Bioss, Beijing, China), CD63 (1:5000, 67605-1-Ig, Proteintech, Wuhan, China), CD81 (1:5000, 27855-1-AP, Proteintech, Wuhan, China), TSG101 (1:5000, 28283-1-AP, Proteintech, Wuhan, China), Calnexin (1:10 000, 10427-2-AP, Proteintech, Wuhan, China) and β-tubulin (1:10 000, MA5-11732, Thermo Fisher Scientific, MA, USA). The membranes were incubated with the primary antibodies overnight at 4 °C. After being incubated for 12 h, membranes were washed three times with TBST and incubated with HRP-conjugated secondary antibodies at room temperature for 1 h. After washing again, the color was visualized using a hypersensitive ECL chemiluminescence reagent (MA0186-1, MeilunBio, Liaoning, China). Signals were captured using a chemiluminescence imaging system. Band intensity was analyzed using ImageJ software, normalized to β-tubulin as the internal reference protein, and relative expression levels of target proteins were calculated relative to the control group.^57^

### 2.9 Immunohistochemical analysis

Mouse colon paraffin sections were deparaffinized in xylene and rehydrated through graded ethanol, followed by heat-induced antigen retrieval.^58^ Subsequently, endogenous peroxidase activity was blocked by incubating with hydrogen peroxide (H_2_O_2_) at room temperature in the dark for 25 min. After washing with PBS, sections were covered uniformly with 3% BSA (4240GR200, BioFroxx, Beijing, China) solution and blocked at room temperature for 30 min.^58^ After removing the blocking solution, sections were incubated overnight at 4 °C with the following primary antibodies: claudin-1 (1:400, GB12032, Servicebio, Wuhan, China), occludin (1:1000, GB151401, Servicebio, Wuhan, China), and zonula occludens-1 (ZO-1;1:500, GB115686, Servicebio, Wuhan, China). After thorough washing with PBS, corresponding species-specific HRP-conjugated secondary antibodies were applied and incubated at room temperature for 50 min.^59^ After washing again, color development was performed using 3,3′-diaminobenzidine (DAB; G1212, Servicebio, Wuhan, China). After color development, sections were counterstained with hematoxylin for 3 min, rinsed in running water, differentiated, blued, and rinsed again.^59^ Finally, sections were dehydrated, cleared, mounted, and observed under a light microscope. Semi-quantitative analysis of target protein expression levels was performed using ImageJ software.

### 2.10 16S rRNA gene sequencing analysis

First, total DNA was extracted from fecal samples and stored at −80 °C. Fecal samples from five mice per group were processed separately, and no pooled fecal samples were used. DNA concentration was measured using a NanoDrop, and quality was checked by 1.2% agarose gel electrophoresis.^60^ Primers targeting the V3–V4 hypervariable region of the bacterial 16S rRNA gene were used for PCR amplification. After quantification, amplification products were pooled in equimolar amounts based on required sequencing depth to construct sequencing libraries. Libraries passing quality control were subjected to paired-end 250 bp sequencing on the Illumina NovaSeq platform.^61^ Raw sequences were quality-controlled, denoised, and clustered using the QIIME 2 DADA2 pipeline or Vsearch software to obtain amplicon sequence variants (ASVs) or operational taxonomic units (OTUs).^62^ At the ASV/OTU level, distance matrices between samples were calculated, and differences in the community structure between groups and their significance were assessed using multivariate statistical methods such as principal coordinate analysis combined with PERMANOVA, ANOSIM, and PERMDISP tests. At the taxonomic composition level, supervised and unsupervised ordination, clustering analysis, and statistical tests like Kruskal–Wallis and Wilcoxon tests were used to identify differential taxa between groups.^63^ Based on the above analyses, corresponding charts were generated and statistically interpreted.

### 2.11 Fecal metabolomics analysis

Fecal metabolomic analysis was performed using untargeted liquid chromatography-tandem mass spectrometry (LC-MS/MS). Fecal samples from five mice per group were individually processed as independent biological replicates. Fresh mouse feces were immediately snap-frozen in liquid nitrogen and stored at −80 °C. Before analysis, samples were thawed on ice and thoroughly mixed. Approximately 50 mg of sample was weighed, and pre-cooled extraction solvent (methanol : acetonitrile : water = 2 : 2 : 1) was added.^64^ Samples were thoroughly homogenized using a tissue homogenizer or bead beating (performed at 4 °C) and then placed at −20 °C for 20 min to precipitate proteins.^65^ After centrifugation at 4 °C, 15 000 g for 15 min, the supernatant was collected, filtered through a 0.22 µm membrane, and injected for analysis.^65^ Chromatographic separation was performed using an ACQUITY UPLC system (Waters, Milford, MA, USA) equipped with an HSS T3 column (150 mm × 2.1 mm, 1.8 µm).^66^ Mass spectrometric detection was performed using a Thermo Orbitrap Exploris 120 mass spectrometer equipped with an electrospray ionization source (BRE725531, Thermo Fisher Scientific, MA, USA). Raw data were processed using Compound Discoverer™ 3.3 software (version 3.3.2.31, Thermo Fisher Scientific, MA, USA) for peak alignment, retention time correction, and peak area extraction.^67^ The extracted data underwent metabolite identification and quantification, data preprocessing, and quality assessment, followed by data analysis and visualization.

### 2.12 Statistical analysis

Statistical analysis was performed using GraphPad Prism software. Data are presented as mean ± standard deviation (SD). Comparisons between two groups were performed using a paired Student's *t*-test. Comparisons among multiple groups were performed using one-way analysis of variance for normally distributed data with homogeneous variance. Spearman's correlation coefficient was used for correlation analysis. For non-normally distributed data, the Kruskal–Wallis test followed by Dunn's multiple-comparison test was applied. *P* values from multiple taxa or metabolite comparisons were adjusted using the Benjamini–Hochberg false discovery rate method where appropriate. Asterisks indicate significance levels: ‘ns’ for not significant, ‘*’ for *P* < 0.05, ‘**’ for *P* < 0.01, and ‘***’ for *P* < 0.001.

## 3 Results

### 3.1 Screening of small molecule combinations and performance verification

**Fig. 1:**
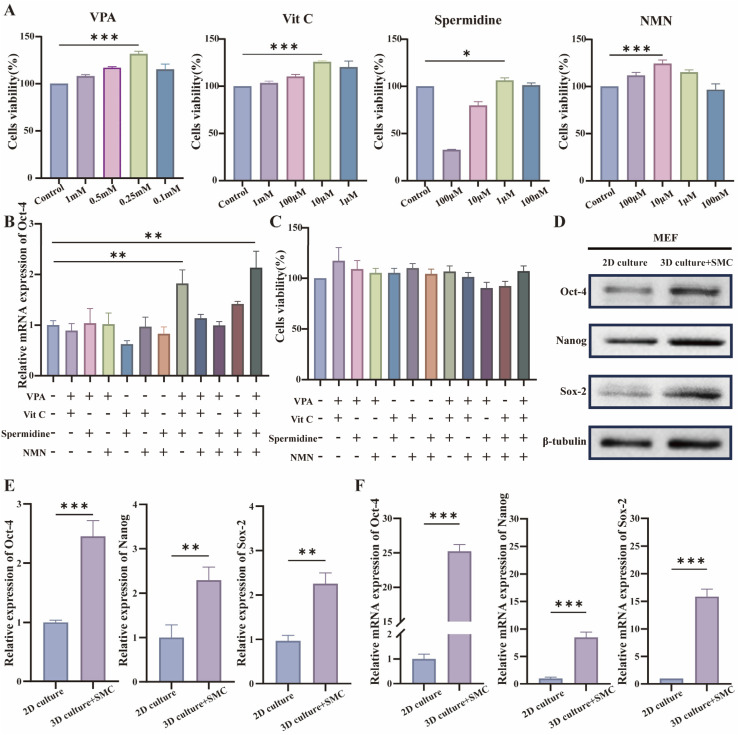
Screening of small-molecule combinations and their effects on stemness-associated markers. (A) Cell viability of MEFs treated with VPA, VitC, spermidine, and NMN at different concentrations. (B) Relative Oct-4 mRNA expression and (C) cell viability in MEFs treated with different combinations of VPA, VitC, spermidine, and NMN (presence/absence is indicated by “+”/“−” below each bar). (D) Representative immunoblot analysis and (E) densitometric quantification of Oct-4, Nanog and Sox-2 in MEFs cultured under conventional 2D conditions or in 3D culture supplemented with SMC. β-tubulin was used as a loading control. (F) RT-qPCR analysis of Oct-4, Nanog and Sox-2 mRNA levels in 2D culture *versus* 3D culture + SMC, expressed relative to 2D culture. Data are presented as mean ± SD from *n* = 3 independent experiments. Statistical significance was analyzed using the *t*-test. ns, not significant. *P < 0.05, **P < 0.01, and ***P < 0.001.

To evaluate the potential impact of small molecule drugs on cell state and identify the optimal combination regimen, this study first assessed four compounds (VPA, VitC, spermidine, and NMN) through single-agent dose-gradient treatment, systematically examining their effects on cell viability. VPA exhibited a dose-dependent effect, with the most significant enhancement of cell viability observed at 0.25 mM; both lower and higher concentrations resulted in diminished effects. VitC consistently improved cell viability across a concentration range of 10 µM to 1 mM and showed the most pronounced enhancement of cell viability at 10 µM. Spermidine displayed a clear concentration dependency, 100 µM treatment significantly suppressed cell viability, while 1 µM treatment slightly promoted it. NMN showed the most pronounced enhancement of cell viability at 10 µM (Fig. 1A). Because the CCK-8 assay reflects cellular metabolic activity and viability, values exceeding 100% may indicate enhanced metabolic activity or a moderate proliferative tendency.

After establishing the tolerable concentration ranges for each drug, we further evaluated the regulatory effects of different small-molecule combinations on the transcription level of the stemness-associated gene Oct-4, while simultaneously monitoring their impact on cell viability. Compared to the untreated control group, most combinations effectively upregulated Oct-4 mRNA expression while maintaining cell viability within 90–120%. Notably, the four-molecule combination (VPA + VitC + Spermidine + NMN) induced the most significant increase in Oct-4 mRNA expression, exceeding a 2-fold elevation, and its effect surpassed that of most dual- or triple-drug combinations (Fig. 1B and C). The results indicated that this composite regimen possesses a strong capacity to activate stemness-associated transcriptional programs without significantly affecting cell survival. Based on these screening results, we applied this small molecule combination (denoted as SMC: VPA + VitC + Spermidine + NMN) to a 3D culture system to further investigate its potential to promote stemness-associated phenotypes. Western blot analysis further confirmed that SMC significantly enhanced the expression of Oct-4, Nanog, and Sox-2 (Fig. 1D and S1). Densitometric quantification showed that Oct-4 expression increased to about 3.5-fold, while Nanog and Sox-2 levels were elevated more than 2-fold (Fig. 1E). RT-qPCR analysis revealed that, compared to the two-dimensional (2D) culture group, cells cultured under 3D conditions and treated with SMC exhibited significantly upregulated mRNA expression of stemness-associated markers, which were upregulated approximately 25-fold (Oct-4), 8-fold (Nanog), and 16-fold (Sox-2), respectively (Fig. 1F). In summary, SMC demonstrated the strongest induction of Oct-4 transcription in the preliminary screen and, under 3D culture conditions, concurrently promoted the transcriptional and translational expression of stemness-associated markers, thereby significantly reinforcing the stemness-associated molecular phenotype. The above results indicated that this SMC holds promise for enhancing stemness-associated marker expression in MEFs, providing an important experimental basis for subsequent functional studies and application development.

### 3.2 SMC promotes eNV production and eNV physicochemical characteristics

**Fig. 2:**
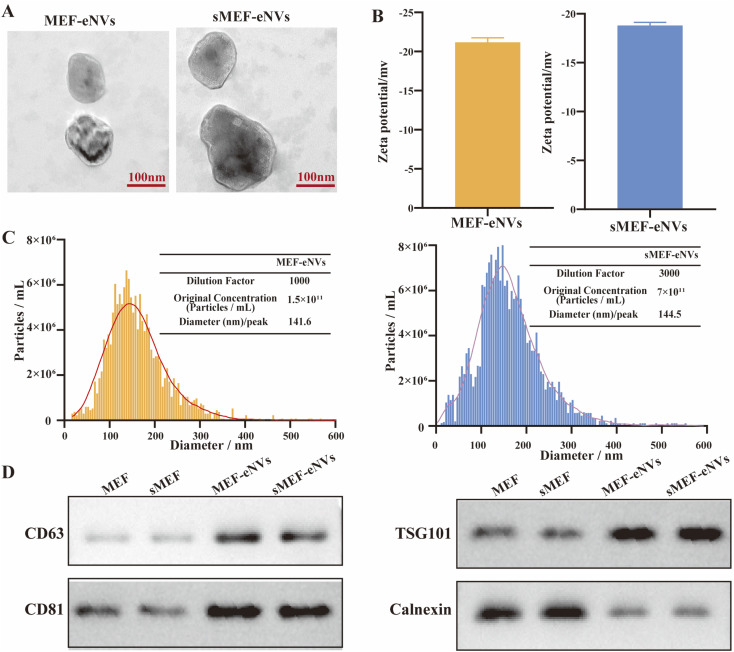
SMC promotes spheroid formation and characterization of MEF-derived extruded nanovesicles. (A) TEM image of MEF-eNVs and sMEF-eNVs. (B) Particle size distribution profiles of MEF-eNVs and sMEF-eNVs. Characterization of each group was performed on samples from a single preparation. Additional batch reproducibility is provided in Fig. S2B. (C) Zeta potential of MEF-eNVs and sMEF-eNVs. Data are presented as mean ± SD from *n* = 3 independent experiments. (D) Western blot analysis of EV-associated markers CD63, CD81, and TSG101 and the negative marker calnexin.

To further explore the effects of the SMC small molecule combination on the 3D culture system, we first assessed its effect on cell aggregation through morphological observation. Fig. S2A shows that after SMC treatment, the cell spheroids formed in the 3D culture system exhibited a denser structure, clearer boundaries, and a significantly higher number compared to the control group. Quantitative analysis further confirmed that the spheroid count in the SMC-treated group increased significantly, demonstrating that the combination effectively promoted 3D spheroid formation and stability. Subsequently, nanovesicles were extracted *via* mechanical extrusion from both naive MEFs and SMC-conditioned MEFs with enhanced stemness-associated marker expression, respectively denoted as MEF-eNVs and sMEF-eNVs, and their physicochemical properties were systematically characterized. TEM images revealed that both vesicle types exhibited typical cup-shaped or spherical exosome-like morphology, with diameters primarily concentrated at around 100 nm (Fig. 2A). Nanoparticle tracking analysis showed that the peak particle sizes of MEF-eNVs and sMEF-eNVs were approximately 141.6 nm and 144.5 nm, respectively. And particle concentration of MEF-eNVs and sMEF-eNVs is about 1.5 × 10^11^ particles per mL and 7 × 10^11^ particles per mL, which indicated that the production of nanovesicles released by cells significantly increased under the SMC-induced stemness-enhanced state (Fig. 2B). Furthermore, zeta potential showed that both vesicle types carried a negative surface charge, consistent with the electrical characteristics of typical extracellular vesicles, supporting their structural integrity and solution stability (Fig. 2C). To further validate the vesicular identity and purity of the prepared nanovesicles, western blot analysis was performed to detect representative EV-associated markers. Both MEF-eNVs and sMEF-eNVs showed positive signals for CD63, CD81, and TSG101, indicating that these mechanically extruded nanovesicles retained typical EV-like protein features. In contrast, the endoplasmic reticulum marker calnexin was abundant in parental cell lysates but was detected at a low level in MEF-eNVs and sMEF-eNVs, suggesting limited contamination by intracellular organelles (Fig. 2D). In this study, sMEF-eNVs were generated using an integrated preparation strategy consisting of 3D culture, small-molecule conditioning, and mechanical extrusion. MEF-eNVs and sMEF-eNVs were prepared using the same extrusion and purification procedures; therefore, the comparison between these two vesicle preparations mainly reflects the influence of donor-cell conditioning on the resulting eNVs under a constant extrusion workflow. These results demonstrated that SMC treatment not only promoted 3D cell spheroid formation but also enhanced the yield of extruded nanovesicles, laying an experimental foundation for further exploring their potential applications for subsequent functional evaluation.

### 3.3 Strain identification and growth characteristic analysis

Although strategies involving EVs can effectively promote intestinal mucosal repair, they are limited in rapidly optimizing a compromised gut microbiota. In contrast, oral probiotic delivery has been demonstrated to directly act on gut microbial ecology and optimize its structure.^44,68,69^*L. rhamnosus* exhibited unique advantages *via* a multi-dimensional synergistic mechanism of “immune modulation–barrier repair–microbiota remodeling”, which can promote Treg differentiation while reducing the infiltration of tissue-damaging CD8^+^ T cells, thereby restoring intestinal immune balance from multiple perspectives, and represents a promising intervention strategy for UC treatment.^70,71^

To ensure the reliability and reproducibility of subsequent experiments, we systematically identified and analyzed the growth characteristics of the *L. rhamnosus* strain used in this study, verifying its reliable origin and well-defined phenotypic traits. After cultivation on solid agar plates, typical *Lactobacillus*-like colonies were observed, with regular edges, smooth surfaces, and uniform distribution. Microscopic examination revealed that the bacterial cells were predominantly short rods with consistent morphology, matching the typical characteristics of the genus *Lactobacillus* (Fig. S3A). Subsequently, a molecular biological approach targeting the conserved 16S rRNA gene was applied, and PCR successfully amplified a distinct, specific band of approximately 1.5 kb (Fig. S3B), consistent with the expected target fragment. The obtained sequence was compared with reference sequences in public databases, and a phylogenetic tree was constructed. Phylogenetic analysis showed that the strain in this study clustered within the clade containing *L. rhamnosus* reference sequences, confirming its identity as *L. rhamnosus* (Fig. S3C). Finally, the growth characteristics of the strain were quantitatively analyzed by monitoring OD600 changes during incubation, and a standard growth curve was generated. The OD600 values displayed a typical sigmoidal (“S-shaped”) growth pattern, including a lag phase, a logarithmic phase, and an eventual stationary phase (plateau). Further analysis revealed a strong linear positive correlation between OD600 values and viable colony counts from plate enumeration (Fig. S3D). This correlation facilitates experimental operation by enabling reliable estimation of viable bacterial concentration through rapid OD600 measurement, thus standardizing bacterial suspension preparation and ensuring consistent and accurate dosing in subsequent intervention assays. In summary, the strain employed in this study was confirmed to be *L. rhamnosus* with well-characterized growth kinetics, establishing a reliable microbiological basis for further in-depth investigations into its specific intervention mechanisms and therapeutic effects against colitis.

### 3.4 Synergistic effect of sMEF-eNVs + *L. rhamnosus* on the DSS-induced UC model

**Fig. 3:**
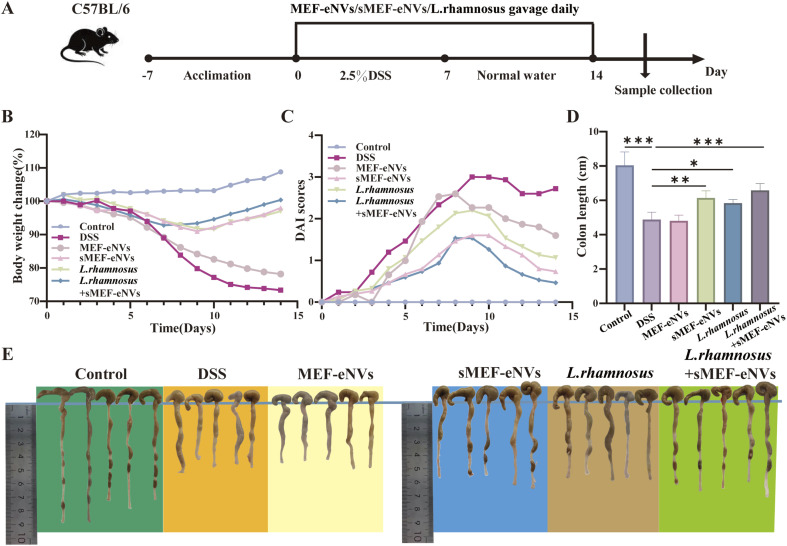
sMEF-eNVs and *L. rhamnosus* alleviate DSS-induced UC in C57BL/6 mice. (A) Experimental scheme of DSS colitis induction and daily gavage intervention (MEF-eNVs, sMEF-eNVs, *L. rhamnosus*, or *L. rhamnosus* + sMEF-eNVs), followed by sample collection on day 14. (B) Body-weight change and (C) DAI scores over time. (D) Colon length measured on day 14. (E) Representative images of excised colons from each group. Data are presented as mean ± SD, *n* = 5 mice per group. Colon length was analyzed using one-way analysis of variance. ns, not significant; *P < 0.05, **P < 0.01, and ***P < 0.001.

Based on our previously identified and characterized *L. rhamnosus*, we further established a 2.5% DSS-induced acute UC model in C57BL/6 mice to evaluate the therapeutic efficacy of *L. rhamnosus* combined with sMEF-eNVs. The experimental design and treatment timeline are illustrated in Fig. 3A. During the modeling period, mice in the DSS model group exhibited typical progressive weight loss, accompanied by a significant increase in the DAI score, confirming successful establishment of the colitis phenotype. In contrast, treatment with either sMEF-eNVs or *L. rhamnosus* alone alleviated the disease phenotype to some extent, manifested as attenuated weight loss and a weakened upward trend in the DAI score. However, the combination of *L. rhamnosus* + sMEF-eNVs exhibited the most significant synergistic improvement. By the end of the experiment, the weights of mice in the *L. rhamnosus* + sMEF-eNVs group returned to levels close to those of the normal control group (Fig. 3B); meanwhile, the DAI score was maintained within a low range (Fig. 3C). Colon shortening is a key macroscopic indicator of colonic damage in colitis.^72^ Compared to the control group, the DSS model group had significantly shortened colons. Treatment with either sMEF-eNVs or *L. rhamnosus* alone partially reversed this shortening, and the *L. rhamnosus* + sMEF-eNVs treatment group exhibited the most prominent improvement, with colon length restored to approximately 6.4 cm (Fig. 3D and E). In summary, comprehensive analysis of multiple key indicators-weight change, DAI score, and colon length-demonstrates that the sMEF-eNVs + *L. rhamnosus* combined intervention strategy showed superior overall ameliorative effects in reducing systemic disease activity and repairing macroscopic colonic damage compared to any single treatment, establishing a phenotypic foundation for subsequent mechanistic exploration.

**Fig. 4:**
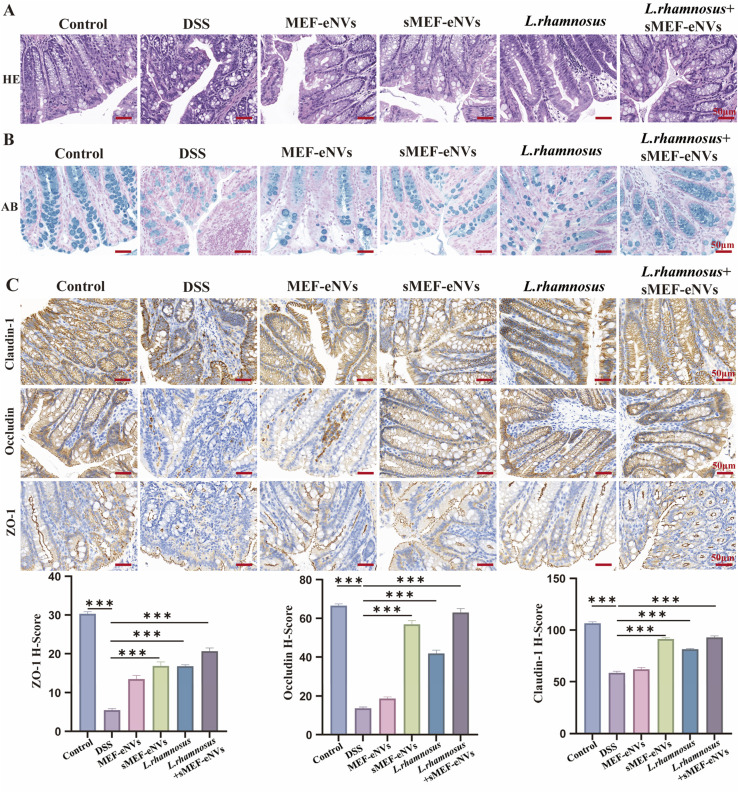
Histological recovery and restoration of intestinal barrier markers following interventions in DSS colitis. (A) Representative H&E staining of colon sections from each group (scale bar: 50 µm). (B) Representative AB staining of colon sections from each group (scale bar: 50 µm). (C) IHC of tight-junction proteins claudin-1, occludin, and ZO-1 (scale bar: 50 µm), with corresponding semi-quantitative H-score analyses. Data are presented as mean ± SD, *n* = 3 mice per group. The H-score was analyzed using one-way analysis of variance. ns, not significant; *P < 0.05, **P < 0.01, and ***P < 0.001.

Subsequently, we performed H&E and AB staining to further evaluate the reparative effects of sMEF-eNVs + *L. rhamnosus* combined intervention on DSS-induced colonic damage at the histopathological level. As shown in Fig. 4A and B, the DSS model group exhibited severe destruction of colonic mucosal architecture, manifested as crypt disorganization and disruption of epithelial continuity. Concurrently, AB staining demonstrated marked thinning of the mucus layer and a decrease in goblet cell numbers, verifying impairment of the mucus barrier. Treatment with MEF-eNVs led to partial restoration of crypt architecture. However, abnormalities including irregular glandular arrangement, focal tissue lesions, and weak AB-positive staining persisted, indicating limited reparative effects. In contrast, sMEF-eNVs intervention more effectively promoted tissue repair, as evidenced by a more regular longitudinal arrangement of the crypts, epithelial integrity was markedly improved, inflammatory infiltration was reduced, AB-positive signals were enhanced, and goblet cell staining was more abundant. The overall morphology approached that of the control group, showing superior performance to MEF-eNVs in alleviating tissue damage and reconstructing the mucus barrier. *L. rhamnosus* alone partially improved histological abnormalities and enhanced mucus staining, but its degree of repair was intermediate between that of MEF-eNVs and sMEF-eNVs. It is noteworthy that the *L. rhamnosus* + sMEF-eNVs group showed the most significant reparative effects in both tissue architecture and the mucus barrier, including the most intact crypt structure, the best-preserved epithelial continuity, the strongest AB-positive signals and the most close resemblance to the normal control distribution. This collectively indicated that the combination strategy is superior to any single intervention in promoting tissue repair and mucus barrier reconstruction.

To quantitatively assess the restoration of epithelial barrier function at the molecular level, we examined the expression of key proteins (claudin-1, occludin, and ZO-1). IHC results showed that, in the control group, the junctional regions between colonic epithelial cells exhibited strong and continuous positive signals. In contrast, signals in the DSS model group were significantly weakened and appeared discontinuous, indicating disruption of the tight junction structure (Fig. 4C). Among all intervention groups, MEF-eNVs had a limited restorative effect on protein expression, with only minor signal enhancement. In comparison, the sMEF-eNVs group showed significantly stronger positive signals with better continuity of distribution within the epithelium. Semi-quantitative H-score analysis verified this, with overall higher scores in the sMEF-eNVs group compared to the MEF-eNVs group, quantitatively confirming its stronger tight junction repair capacity. At the transcriptional level, RT-qPCR results showed that mRNA expression of claudin-1, occludin, and ZO-1 was significantly downregulated in the DSS model group (Fig. S4). MEF-eNVs intervention failed to effectively reverse this trend, whereas sMEF-eNVs intervention significantly upregulated the expression of all three genes, with levels higher than those in the MEF-eNVs group. Importantly, the *L. rhamnosus* + sMEF-eNVs group exhibited the strongest restorative effects at both protein and gene levels. IHC staining showed the strongest signal intensity and optimal continuity of distribution, while mRNA expression levels were also upregulated most significantly. Collectively, these findings demonstrate that sMEF-eNVs effectively restore intestinal tight junctions at both transcriptional and translational levels and exert synergistic barrier-enhancing effects when combined with *L. rhamnosus*, thereby reinforcing epithelial barrier integrity at the molecular level.

### 3.5 Synergistic remodeling of intestinal immune homeostasis by sMEF-eNVs and *L. rhamnosus* in a DSS-induced UC model

**Fig. 5:**
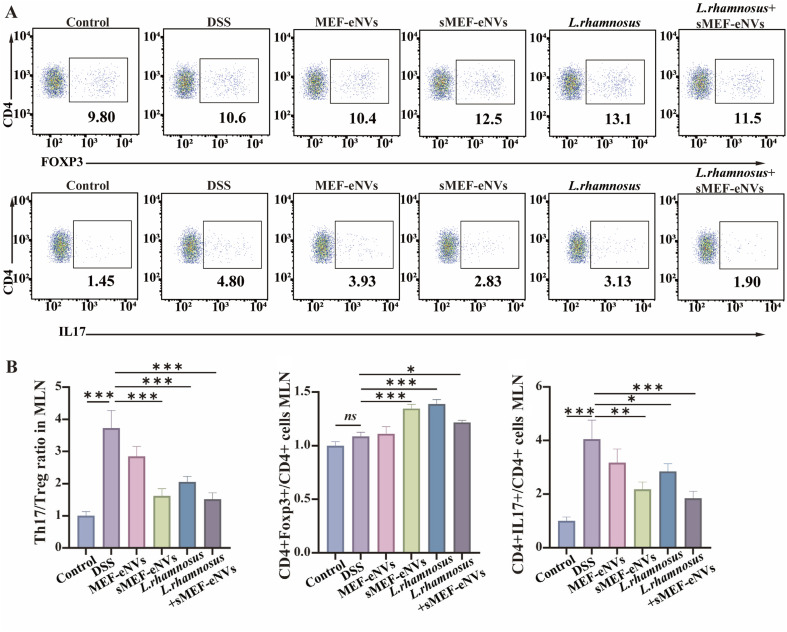
Combined treatment with *Lactobacillus rhamnosus* and sMEF-eNVs restores the Th17/Treg balance in mesenteric lymph nodes of DSS-induced colitis mice. (A) Representative flow-cytometry plots showing CD4^+^FOXP3^+^ regulatory T (Treg) cells and CD4^+^IL-17^+^ T helper 17 (Th17) cells in mesenteric lymph nodes (MLNs) from the Control, DSS, MEF-eNVs, sMEF-eNVs, *L. rhamnosus*, and *L. rhamnosus* + sMEF-eNVs groups. The values shown within the gates indicate the percentages of FOXP3^+^ or IL-17^+^ cells among CD4^+^ T cells. (B) Quantitative analysis of the Th17/Treg ratio, the proportion of CD4^+^FOXP3^+^ Treg cells among CD4^+^ T cells, and the proportion of CD4^+^IL-17^+^ Th17 cells among CD4^+^ T cells in MLNs. Data are presented as the mean ± SD; *n* = 3 mice per group. Flow-cytometric data were analyzed using one-way analysis of variance. ns, not significant; *P < 0.05, **P < 0.01, and ***P < 0.001.

To elucidate the immunomodulatory mechanism of sMEF-eNVs + *L. rhamnosus* intervention, we performed flow cytometric analysis of CD4^+^ T cell subsets in MLNs. As shown in Fig. 5A and S5A, DSS treatment markedly increased the proportion of Th17 cells (4.80%), significantly higher than that in the control group (1.45%), indicating that the DSS-induced murine colitis model significantly disrupted intestinal immune balance. Intervention with MEF-eNVs reduced the Th17 proportion somewhat (3.93%), while sMEF-eNVs caused a greater reduction (2.83%). The combination group further decreased it to 1.90%, approaching normal levels. Quantitative analysis revealed that DSS treatment significantly elevated the Th17/Treg ratio. All intervention groups reduced this ratio, with sMEF-eNVs showing a better effect than MEF-eNVs and the combination therapy group achieving the lowest ratio, which indicated that sMEF-eNVs more effectively corrected the DSS-induced Th17-dominant state, and the combination strategy had an enhanced effect on reshaping immune balance. Regarding the regulation of Treg (CD4^+^Foxp3^+^) cells, the Treg proportion in the DSS group (10.6%) showed no significant increase compared to the control (9.80%), suggesting that its immunosuppressive function might be insufficient to counter the strong Th17 response. Among intervention groups, the *L. rhamnosus* alone group had the highest Treg proportion (13.1%), the sMEF-eNVs alone group also showed an increase (12.5%), and the proportion in the combination group was 11.5%. In summary, the core advantage of *L. rhamnosus* + sMEF-eNVs combination intervention is not a singular, massive increase in Tregs, but rather a significant suppression of pro-inflammatory Th17 cells coupled with a synchronous optimization of the critical Th17/Treg immune balance ratio. These results indicated that sMEF-eNVs + *L. rhamnosus* can synergistically reshape the immune response landscape in the UC model, shifting it from a hyper-inflammatory state towards immune tolerance, thereby creating a more favorable immune microenvironment for mucosal repair.

**Fig. 6:**
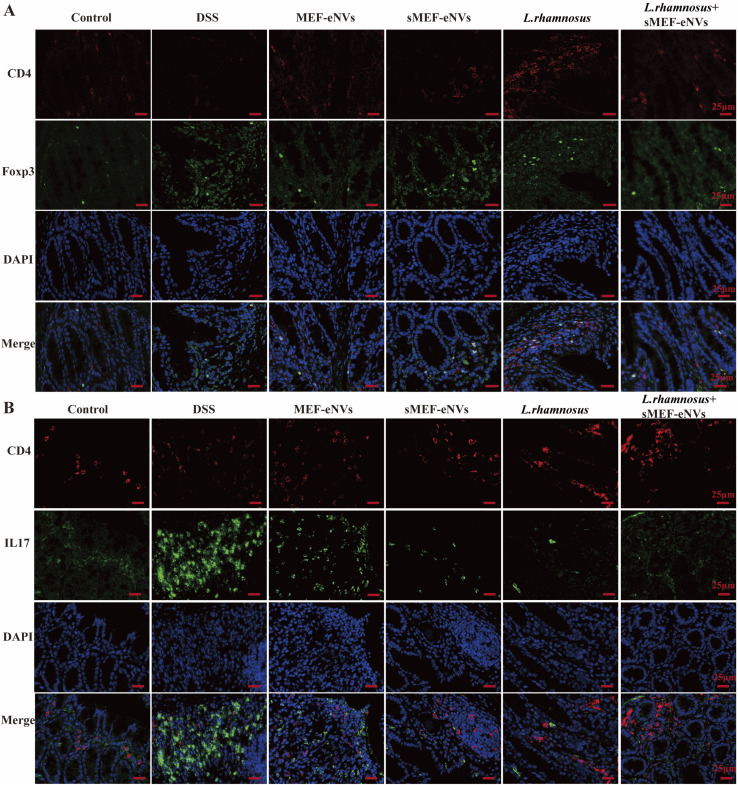
Immunofluorescence analysis of Treg and Th17 cell infiltration in the colonic tissues of DSS-induced colitis mice. Representative immunofluorescence images of colon sections from the Control, DSS, MEF-eNVs, sMEF-eNVs, *Lactobacillus rhamnosus*, and *L. rhamnosus* + sMEF-eNVs groups. (A) Co-immunofluorescence staining of CD4 and FOXP3 for the identification of CD4^+^FOXP3^+^ regulatory T (Treg) cells. CD4 is shown in red, FOXP3 in green, and nuclei counterstained with DAPI in blue. (B) Co-immunofluorescence staining of CD4 and IL-17 for the identification of CD4^+^IL-17^+^ T helper 17 (Th17) cells. CD4 is shown in red, IL-17 in green, and nuclei in blue. Merged images show the spatial distribution and colocalization of the indicated markers in colonic tissues. *n* = 3 mice per group. Scale bars, 25 µm.

Immunofluorescence results further verified that only a small number of sparsely distributed CD4^+^FOXP3^+^ cells were visible in the control group, with extremely low numbers of CD4^+^IL-17^+^ cells, reflecting the baseline immunoregulatory status under physiological conditions. After DSS treatment, inflammatory infiltration within the tissue was significantly enhanced. The number of CD4^+^IL-17^+^ cells and their IL-17 fluorescence intensity were markedly increased, accompanied by a compensatory increase in CD4^+^FOXP3^+^ cells, illustrating the coexistence of an enhanced Th17 response and compensatory Treg-cell activation in the inflammatory environment. Compared with the DSS group, the MEF-eNVs group showed no significant anti-inflammatory effect. In contrast, both the sMEF-eNVs group and *L. rhamnosus* group effectively reduced the infiltration level of CD4^+^IL-17^+^ cells and increased the relative abundance of CD4^+^FOXP3^+^ cells, indicating that both treatments possess immunomodulatory activity. sMEF-eNVs + *L. rhamnosus* intervention exhibited the most significant improvement. Infiltration of Th17-like cells was further reduced, and Treg-related signals were noticeably restored, collectively shifting the immune microenvironment from a pro-inflammatory toward a regulatory state. This result was consistent with the trend of improved Th17/Treg balance suggested by flow cytometry analysis, further supporting the synergistic role of the combined intervention in regulating intestinal immune balance (Fig. 6A and B).

To dissect the mechanism of immune remodeling at the molecular level, we further examined the transcription levels of key cytokines in colon tissue. Fig. S5B shows that the combined intervention of sMEF-eNVs + *L. rhamnosus* synergistically regulates pro-inflammatory and anti-inflammatory signals, optimizing the inflammatory microenvironment. DSS induction significantly elevated mRNA levels of the pro-inflammatory cytokines IL-6 and TNF-α. Following intervention, MEF-eNVs treatment resulted in a slight decrease in IL-6 and TNF-α expression, but levels remained relatively high. In contrast, sMEF-eNVs intervention significantly downregulated the expression of both factors, demonstrating a clearly superior effect in suppressing the pro-inflammatory response compared to MEF-eNVs. Notably, the inhibitory effect of *L. rhamnosus* + sMEF-eNVs was most prominent, reducing IL-6 and TNF-α expression to the lowest levels among all intervention groups. This finding corroborates the previously observed phenotypes of reduced histological damage and decreased Th17 proportion, confirming the synergistic advantage of the combination therapy in suppressing excessive inflammation. Concurrently, analysis of immunomodulatory/anti-inflammatory factors revealed that all interventions upregulated the expression of IL-10 and TGF-β to varying degrees. The extent of upregulation for both cytokines in the sMEF-eNVs group was generally higher than that in the MEF-eNVs group. The combination intervention group showed the most significant upregulation, with TGF-β expression reaching the highest level among all groups.

The above findings, together with the Th17/Treg flow cytometry results, form a coherent immunoregulatory logical chain. Specifically, *L. rhamnosus* + sMEF-eNVs combination therapy not only effectively suppresses the pro-inflammatory signals (IL-6 and TNF-α) driven by Th17 cell responses, but also potently activates the immunomodulatory axis (IL-10 and TGF-β) associated with Treg function. This “bidirectional regulation” of both sides of the inflammatory balance enables it to exhibit comprehensive potential superior to any single intervention strategy in reshaping the colitic immune microenvironment and promoting the restoration of mucosal homeostasis.

### 3.6 Regulation of gut microbiota in UC mice by *L. rhamnosus* + sMEF-eNVs

**Fig. 7:**
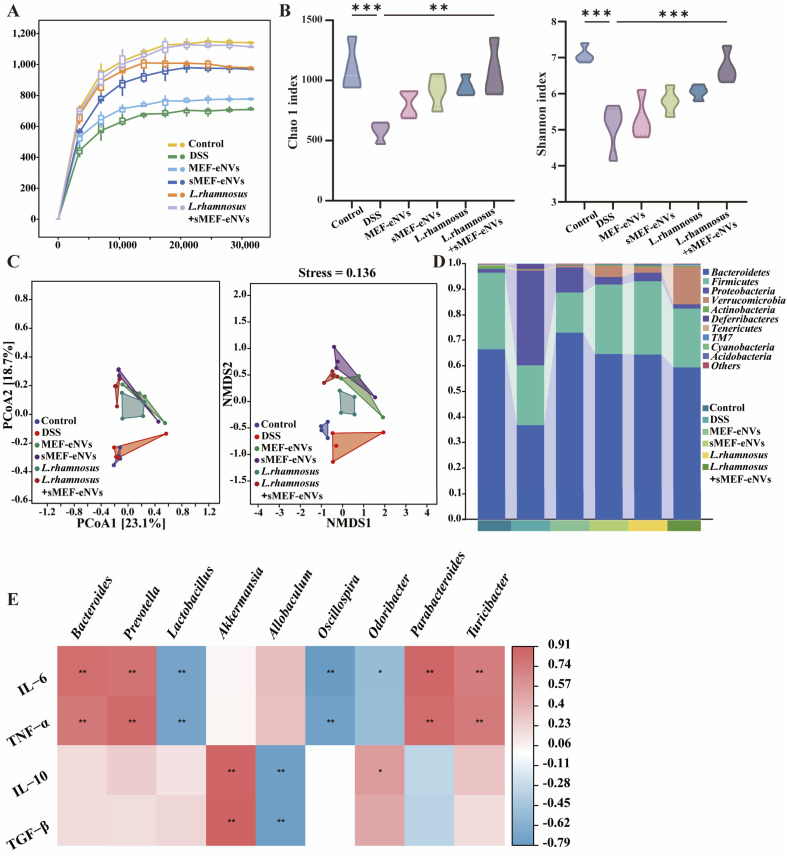
Gut microbiota alteration following intervention in DSS colitis. (A) Rarefaction curves showing sequencing depth *versus* observed community richness. (B) α-Diversity indices (Chao1 and Shannon) displayed as violin plots. (C) β-diversity analysis by PCoA and NMDS ordination (stress value shown). (D) Stacked bar plots of phylum-level relative abundance. (E) Spearman correlation heatmap between key genera and cytokine expression. *n* = 4 fecal samples per group. Alpha diversity was analyzed using the Kruskal–Wallis test followed by Dunn's test. *P* values for differential taxa were adjusted using the Benjamini–Hochberg FDR method where appropriate. ns, not significant; *P < 0.05, **P < 0.01, and ***P < 0.001.

During the progression of UC, the inflammatory microenvironment in the colon can lead to severe gut microbiota dysbiosis, characterized by the overgrowth of pathogenic bacteria and the depletion of beneficial anaerobes. This imbalance further exacerbates colonic inflammation. Therefore, correcting dysbiosis and restoring microbial homeostasis is a key therapeutic strategy for UC. The above results illustrated that the combined application of *L. rhamnosus* + sMEF-eNVs can effectively alleviate clinical symptoms in UC mice, enhance intestinal barrier function, and modulate immune responses. These significant therapeutic effects encouraged us to further analyze the regulatory impact of the combination on the gut microbiota to investigate whether its efficacy is associated with the remodeling of the microbial community. To this end, this study employed 16S rRNA gene high-throughput sequencing to investigate the effects of *L. rhamnosus* + sMEF-eNVs intervention on the composition of the gut microbiota in DSS-induced UC mice, aiming to reveal the underlying therapeutic mechanism at the microbiome level. Fig. 7A shows that, as sequencing depth increased, the rarefaction curves for all samples rose rapidly and reached a plateau, indicating that the sequencing depth in this study was sufficient to capture the microbial diversity within the samples and the data are suitable for subsequent community structure analysis. Analysis of alpha diversity revealed that compared to the model group and other intervention groups, the combination treatment group showed a significant increase in both species richness and community evenness (Fig. 7B). This suggests that *L. rhamnosus* + sMEF-eNVs combined intervention not only increases the species number but also improves the community structure, thereby comprehensively inhibiting the loss of microbial diversity induced by colitis. Given that higher microbial diversity is closely associated with gut homeostasis and host health, this result provides microbiological evidence for the benefit of the combination therapy. Furthermore, inter-group diversity (beta-diversity) of the gut microbiota was analyzed using Principal Coordinates Analysis (PCoA) and Non-Metric Multidimensional Scaling (NMDS). PCoA results clearly demonstrated the clustering of gut microbiota among different groups. In the PCoA plot (Fig. 7C), sample points clustered using the *L. rhamnosus* + sMEF-eNVs combination treatment group and the DSS model group showed the most distinct separation along the PC1 axis (explaining 24.7% of the variance), suggesting fundamental differences in their microbiota structure. More importantly, sample points from the combination treatment group overlapped with the distribution area of the healthy control group, indicating that its microbiota composition had shifted towards a healthy state. NMDS analysis yielded similar conclusions (stress value = 0.162), further supporting the robustness of the results. These data collectively demonstrated that the combination of *L. rhamnosus* and sMEF-eNVs significantly alleviated gut microbiota dysbiosis in the DSS-induced UC model and was more effective than either treatment alone.

Phylum-level analysis revealed that the gut microbiota composition was markedly altered in the DSS-induced UC model. The abundance of *Firmicutes* was significantly decreased, whereas *Proteobacteria* exhibited abnormal overgrowth, directly leading to a reduced *Firmicutes*/*Proteobacteria* ratio, a well-established indicator of gut dysbiosis. However, the combination therapy with *L. rhamnosus* and sMEF-eNVs successfully reversed this trend, shifting the microbiota composition back towards a healthy state (Fig. 7D). In contrast, the individual application of either component failed to effectively correct this imbalance, suggesting a synergistic effect between the two in regulating microbial homeostasis. Subsequent in-depth analysis at the genus level revealed specific bacterial genus markers of dysbiosis. Fig. S6 shows that in the DSS-treated group, the abundance of a series of potentially pathogenic or inflammation-associated genera (such as *Bacteroides*, *Prevotella*, *Parabacteroides*, *Turicibacter*, *etc.*) increased, while the abundance of beneficial genera with anti-inflammatory or SCFA-producing functions (such as *Lactobacillus* and *Oscillospira*) decreased. The combined intervention with *L. rhamnosus* + sMEF-eNVs may reshape these key genera, indicating that it may exert its therapeutic effect by modulating critical functional taxa. To further elucidate the association between microbiota and inflammation, Spearman correlation analysis found that the levels of pro-inflammatory cytokines IL-6 and TNF-α were positively correlated with the abundance of genera like *Bacteroides* and *Prevotella* and negatively correlated with the abundance of genera like *Lactobacillus* and *Oscillospira*. Meanwhile, the anti-inflammatory cytokines IL-10 and TGF-β were positively correlated with the abundance of *Akkermansia* (Fig. 7E). These results indicated that *L. rhamnosus* + sMEF-eNVs may alleviate UC by restoring the relative abundance of beneficial bacteria and suppressing the growth of pathogenic bacteria.

### 3.7 Impact of *L. rhamnosus* + sMEF-eNVs synergistic intervention on the fecal metabolome of UC mice

**Fig. 8:**
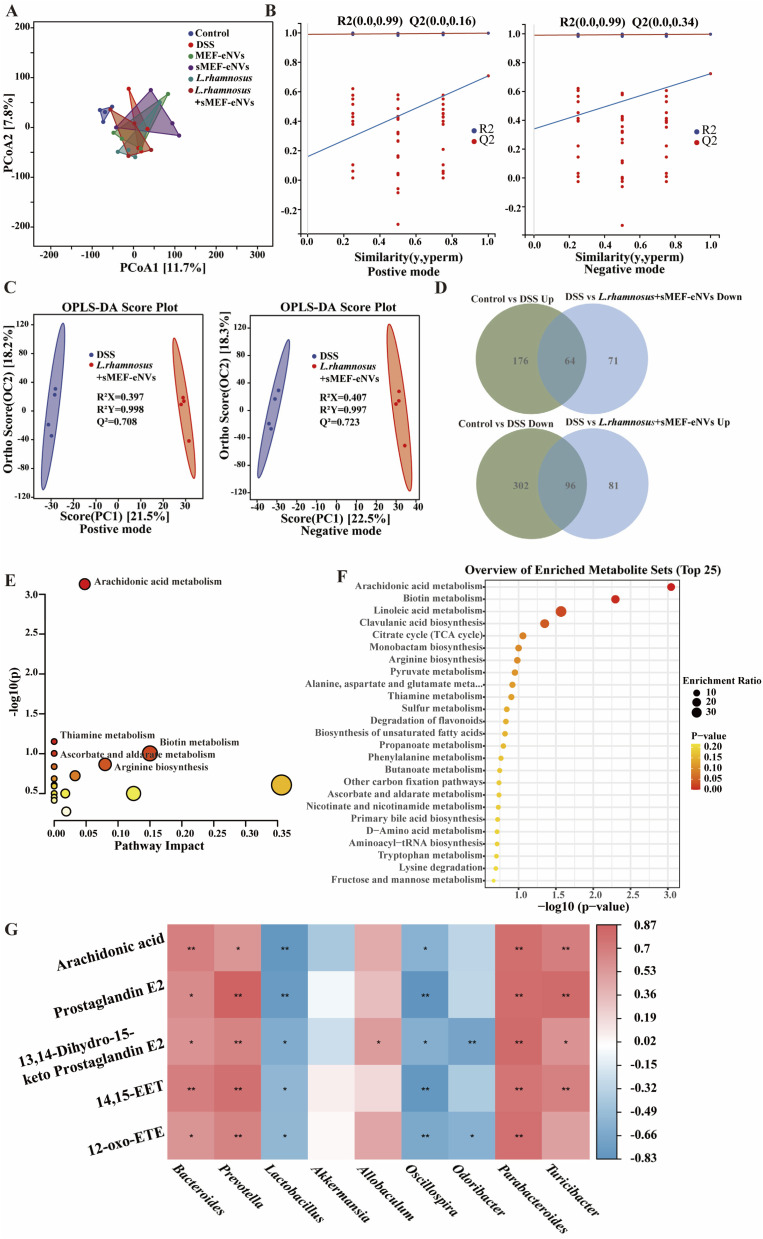
Untargeted metabolomics profiling and pathway enrichment associated with combined therapy. (A) Multivariate ordination of metabolomic profiles among groups. (B) Permutation tests assessing OPLS-DA model robustness in positive and negative ion modes (*R*^2^ and *Q*^2^). (C) OPLS-DA score plots comparing DSS and *L. rhamnosus* + sMEF-eNVs groups in positive/negative modes (*R*^2^*X*, *R*^2^*Y*, and *Q*^2^ shown). (D) Venn diagrams of differential metabolites shared between “Control *vs.* DSS” and “DSS *vs. L. rhamnosus* + sMEF-eNVs” comparisons. (E) Pathway topology analysis (pathway impact *vs.* −log 10(*P*)). (F) Overview of enriched metabolite sets (top 25) presented as a bubble plot. (G) Spearman correlation heatmap between arachidonic-acid-related metabolites and representative bacterial genera. *n* = 4 fecal samples per group. Differential metabolites were screened using fold-change and statistical criteria, followed by Benjamini–Hochberg FDR correction where appropriate. ns, not significant; *P < 0.05, **P < 0.01, and ***P < 0.001.

To systematically elucidate the potential impact of *L. rhamnosus* + sMEF-eNVs synergistic intervention on the metabolic phenotype of UC mice, we conducted untargeted fecal metabolomic analysis. Principal component analysis (PCA) showed that the metabolite profile of the *L. rhamnosus* + sMEF-eNVs group was closer to that of the healthy control group compared to the DSS model group, suggesting a trend towards reversing metabolic disturbances (Fig. 8A). Subsequently, we performed a detailed inter-group comparison using supervised Orthogonal Partial Least Squares Discriminant Analysis (OPLS-DA). Permutation tests of the model comparing the *L. rhamnosus* + sMEF-eNVs group with the DSS group indicated that the model was reliable and not overfitted (Fig. 8B). OPLS-DA showed tight clustering of samples within groups, indicating high similarity of metabolites within the same group. The *L. rhamnosus* + sMEF-eNVs group and the DSS group were distinctly separated into two clusters. The statistical parameters were *R*^2^*X* = 0.397, *R*^2^*Y* = 0.998, and *Q*^2^ = 0.708 in positive-ion mode and *R*^2^*X* = 0.407, *R*^2^*Y* = 0.997, and *Q*^2^ = 0.723 in negative-ion mode, which confirmed highly significant differences in metabolic profiles between the groups (Fig. 8C).

Untargeted fecal metabolomic analysis further quantified the degree of metabolic perturbation. A total of 638 differentially expressed metabolites were identified between the DSS model group and the control group. We identified 312 differential metabolites between the *L. rhamnosus* + sMEF-eNVs group and the DSS group (Fig. 8D). Venn-diagram analysis of the pattern of *L. rhamnosus* + sMEF-eNVs therapy showed that 64 metabolites that were abnormally elevated in the DSS group were significantly reduced, while 96 metabolites suppressed in the DSS group were restored. Kyoto Encyclopedia of Genes and Genomes (KEGG) pathway enrichment analysis of these differential metabolites indicated that the arachidonic acid (AA) metabolism pathway was the most significantly enriched (Fig. 8E and F). This pathway is a core regulatory pathway for inflammatory responses. This finding, at the metabolic level, suggests that the synergistic alleviation of UC by *L. rhamnosus* + sMEF-eNVs may be related to the regulation of arachidonic acid metabolism and its downstream inflammatory mediators. These changes indicate a shift toward a less inflammatory metabolic profile; however, they do not establish direct regulation of the arachidonic acid pathway by the combined treatment.

To validate whether *L. rhamnosus* + sMEF-eNVs synergistic intervention improves the intestinal microenvironment by modulating the AA metabolic pathway, we analyzed related lipid mediators. Fig. S7B shows that the levels of AA and its downstream metabolites PGE2, 13,14-dihydro-15-keto PGE2, 12-oxo-ETE, and 14,15-EET were significantly elevated in the DSS model group. The combination treatment effectively reversed this trend, markedly reducing these pro-inflammatory mediators. These metabolite changes reflect the following metabolic process: under inflammatory stimulation, AA is released from membrane phospholipids and metabolized *via* the COX, LOX, and CYP enzyme systems, respectively (Fig. S7A). The concurrent elevation of PGE2 and its terminal metabolite 13,14-dihydro-15-keto PGE2 in the DSS group indicates intensified synthesis and degradation turnover, continuously driving inflammation. The reduction in all branch products after combination treatment signifies a systemic attenuation of the inflammatory state.

Under inflammatory stimulation, phospholipases such as cPLA2α more readily release AA from membrane phospholipids. The released AA subsequently enters three major metabolic branches, the COX, 12-LOX, and CYP pathways. Through the COX-1/2 pathway, AA is converted to PGG2/PGH2 and enters the PGE2 axis, where it can be further metabolized *via* the 15-PGDH and PTGR1/2 enzymatic cascade to form 13,14-dihydro-15-keto PGE2. Alternatively, AA can be metabolized by 12-LOX/ALOX12 to generate 12-HpETE/12-HETE, which is subsequently converted to 12-oxo-ETE through the action of enzymes including 12-HEDH. A third metabolic branch involves CYP2C/2J enzymes that convert AA to 14,15-EET, which is then hydrolyzed by sEH/EPHX2 to form DHET. The concurrent significant elevation of PGE2 and its downstream metabolite 13,14-dihydro-15-keto PGE2 in the DSS group indicated enhanced turnover in both the synthesis and degradation pathways of PGE2, suggesting persistent inflammatory activity in this group. The increased levels of other AA metabolic branch products further support the presence of ongoing inflammation. In contrast, the combination treatment group showed decreased levels of both AA and its metabolites, demonstrating substantial attenuation of the inflammatory response. The correlation heatmap analysis between gut microbiota and metabolites revealed distinct patterns: genera including *Bacteroides*, *Prevotella*, *Parabacteroides*, and *Turicibacter* tended to exhibit positive correlations with AA and its downstream lipid mediators. Conversely, the genera *Lactobacillus* and *Oscillospira* predominantly showed negative correlations with these inflammatory metabolites (Fig. 8G). These results indicated that *Lactobacillus* and *Oscillospira* played central roles in the therapeutic intervention involving *L. rhamnosus* + sMEF-eNVs.

## 4 Discussion

This study goes beyond traditional single-target interventions and proposes a novel temporally sequential and synergistic therapeutic paradigm: sMEF-eNVs first repair the mucosa and remodel the microenvironment, thereby supporting colonization of *L. rhamnosus*, synergistically restoring microbial and metabolic homeostasis. This synergistic strategy of “preparing the environment first, then introducing the bacteria” mechanistically explains why combined intervention is superior to any single therapy.^73^

The efficacy of probiotics in UC is often limited by a thinned mucus layer, insufficient adhesion sites, and severe local inflammation, resulting in high colonization resistance. Improving the colonization niche rather than increasing bacterial dosage is critical.^74–76^ In this study, sMEF-eNVs repaired mucosal damage, restored tight-junction proteins (ZO-1/occludin/claudin-1), and promoted mucus secretion, creating a low-inflammatory, adhesion-permissive niche for *L. rhamnosus*. The superior therapeutic effect arose not from simple additivity, but from sMEF-eNVs-mediated niche optimization and sustained microbial and metabolic regulation by probiotics in a hospitable environment.^76–78^

Th17/Treg imbalance is a core immunopathological feature of UC [4]. DSS induction elevated the Th17 proportion (to 4.80%) with increased IL-6 and TNF-α, while combined intervention reduced Th17 to ∼1.90%, normalized the Th17/Treg ratio, and allowed homeostatic rebound of Treg cells as inflammation resolved. These results align with studies showing that EVs and lactobacilli alleviate colitis *via* Th17/Treg regulation.^78^ In this study, the *L. rhamnosus* alone group showed the highest Treg proportion, whereas the combination group showed stronger reduction of Th17 cells and the lowest Th17/Treg ratio. This suggests that the therapeutic advantage of the combination treatment may not depend on maximal Treg expansion alone. Instead, *L. rhamnosus* may preferentially support regulatory immune responses, whereas sMEF-eNVs may contribute more strongly to suppression of Th17-associated inflammation and barrier repair, thereby improving the overall immune balance.

16S rRNA sequencing revealed that combination therapy restored α-diversity and shifted microbial structure toward that of healthy controls, correcting DSS-induced dysbiosis in a manner correlated with inflammatory cytokine levels, supporting dysbiosis as an inflammatory driver.^79,80^ Metabolomics identified 312 differential metabolites between the combination and DSS groups, with AA metabolism as the most significantly enriched pathway. AA and downstream eicosanoids amplify mucosal inflammation *via* COX/LOX/CYP cascades. Although pathway enrichment analysis showed significant changes in arachidonic acid-related metabolites, including reduced PGE2 and other inflammation-associated lipid mediators in the combination group, the current data do not establish whether these changes were directly induced by *L. rhamnosus* plus sMEF-eNVs. Fecal metabolomic profiles represent the integrated consequences of host inflammation, intestinal barrier status, immune activity, microbial metabolism, and dietary exposure. Therefore, the reduction of AA metabolites may reflect either direct or indirect modulation of eicosanoid metabolism or, alternatively, a secondary metabolic consequence of reduced AA inflammation and restored intestinal homeostasis. Targeted lipidomics and validation of key enzymes involved in the COX, LOX, and CYP pathways will be required to clarify the causal role of arachidonic acid metabolism in the therapeutic response.

Despite the promising results, this study has several limitations. First, the sample size in each experimental group (*n* = 5) is relatively small. Although a sample size of five animals per group is commonly used in DSS-induced colitis studies and sufficient to detect the pronounced therapeutic effects observed here, it may limit the statistical power to identify subtle differences among intervention groups and increase the risk of type II error. Second, the DSS-induced acute colitis model, while widely accepted, does not fully recapitulate the chronic relapsing nature of human UC, nor does it reflect the genetic heterogeneity and environmental complexity of human disease. The relatively short duration of the experiment also precludes assessment of the long-term safety and sustained efficacy of sMEF-eNVs combined with *L. rhamnosus*. Third, the study lacked a more detailed dose–response analysis for both sMEF-eNVs and *L. rhamnosus*, which would help optimize the combination regimen. Fourth, potential bias related to the lack of blinding in investigator assessments was not addressed in detail. Another limitation of this study is that the individual contributions of 3D culture, small-molecule conditioning, and the extrusion procedure were not fully dissected. Although MEF-eNVs and sMEF-eNVs were generated using identical extrusion and purification procedures, allowing comparison of vesicles derived from differently conditioned donor cells, the current design does not independently determine the extent to which 3D culture or the small-molecule combination contributed to the observed changes in vesicle yield, vesicle cargo, and therapeutic efficacy. Future studies use a factorial design, including 2D-cultured MEFs, 3D-cultured MEFs, 2D MEFs treated with the small-molecule combination, and 3D-cultured MEFs treated with the small-molecule combination. In addition, naturally secreted EVs and mechanically extruded nanovesicles obtained from the same donor-cell conditions should be compared to distinguish the contribution of the extrusion procedure itself. Such experiments will be necessary to define the contribution of each preparation parameter. Finally, the precise molecular cargo within sMEF-eNVs responsible for these effects remains to be fully elucidated. Quantitative proteomics, small-RNA sequencing, and lipidomics will be used to compare the cargo profiles of MEF-eNVs and sMEF-eNVs.

Future studies should validate these findings in larger cohorts and chronic colitis models, optimize the dose and long-term treatment regimen under rigorously blinded conditions, dissect the individual contributions of 3D culture, small-molecule conditioning, and extrusion, and identify the specific bioactive cargo of sMEF-eNVs and molecular mechanisms underlying the therapeutic effects of sMEF-eNVs combined with *L. rhamnosus*. Moreover, strain-specific qPCR, fluorescent labelling, mucosal tissue localisation, quantitative bacterial culture, and persistence experiments will be used to directly determine the abundance, spatial distribution, and persistence of the administered *L. rhamnosus* strain.

## 5 Conclusion

In this study, we used SMC combined with 3D culture to enhance the stemness-associated markers of MEFs. Subsequently, these stemness-enhanced MEFs were used to prepare morphologically uniform, high-yield sMEF-eNVs *via* membrane extrusion. In a 2.5% DSS-induced mouse model of UC, the combined intervention with sMEF-eNVs and *L. rhamnosus* not only upregulated the expression of tight junction proteins, promoted intestinal mucosal repair, reshaped gut microbial diversity, and restored normal intestinal physiological function, but also regulated the Th17/Treg ratio, suppressed the release of pro-inflammatory factors, and thereby re-established intestinal immune homeostasis. Collectively, this study proposes a novel therapeutic strategy based on the synergistic effect of “vesicle-probiotics”, which exerts synergistic therapeutic effects at multiple levels through tissue repair, microbial regulation, and immune modulation, to simultaneously remodel the mucosal microenvironment and maintain immune-microbial homeostasis. These findings provide new experimental evidence and a potential theoretical framework for the combination of extracellular vesicle therapy and microbial therapy in the treatment of UC.

## Ethical approval

All experimental procedures were approved by the Institutional Animal Care and Use Committee of the Medical College of Jiangsu University (Approval No: UJS-IACUC-AP-2022030272).

## Consent for publication

Each co-author has read the manuscript and approved its submission.

## Author contributions

Xinyu Cao, Chao Yan and Qing Ji are responsible for the experimental operations, data collection and analysis. Yutong Pan and Wencheng Shi are responsible for data collection and analysis. Huijun Zhao and Aihua Gong are responsible for data processing and image generation. Zhengzou Fang is responsible for the experimental design and writing of the manuscript. All authors read and approved the final manuscript.

## Conflicts of interest

The authors declared that they have no competing financial interests or personal relationships that could have appeared to influence the work reported in this paper.

## Supplementary Material

NA-OLF-D6NA00298F-s001

## Data Availability

All data generated or analyzed during this study are included in this published article and its supplementary information (SI) files. Supplementary information: additional experimental details, characterization data of sMEF-eNVs, supplementary figures and tables, and supporting data from animal experiments, microbiome analysis, and metabolomic analysis. See DOI: https://doi.org/10.1039/d6na00298f.
