# Supplementary material for: Stemness-associated MEF-derived extruded nanovesicles cooperate with Lactobacillus rhamnosus to alleviate DSS-induced colitis through mucosal microenvironment remodeling
Source: Nanoscale Adv. 2026 Jul 21;8(18):5235–55. doi: 10.1039/d6na00298f (PMC13445723; doi:10.1039/d6na00298f)
Supplement: Supplementary file 1 [file NA-008-D6NA00298F-s001.pdf]

Supplementary Information (SI) for Nanoscale Advances.
This journal is © The Royal Society of Chemistry 2026

Supplement Information

Stemness-associated MEF-derived extruded nanovesicles cooperate with Lactobacillus rhamnosus to alleviate DSS-induced colitis through mucosal microenvironment remodeling

Xinyu Cao,[1*] Chao Yan,[2*] Qing Ji,[1] Yutong Pan,[1] Wencheng Shi,[1] Huijun Zhao,[1] Aihua Gong,[1] Zhengzou Fang[1#]

1. Department of Laboratory Medicine, Affiliated Hospital of Jiangsu University, Zhenjiang, 212001, China.
2. The Affiliated Huai'an Hospital of Xuzhou Medical University and The Second People's Hospital of Huai'an, No. 62, Huaihai Road (S.), Huai'an, 223002, China.

"*" These authors contributed equally to this work

Corresponding author email: Chao Yan (y13852034764@163.com) and myfangzz@163.com (Zhengzou Fang)

2. Methods and Materials

2.1 Materials and reagents

The key small molecules and pharmacological agents used in this study included valproic acid (VPA; S161023, Aladdin, Shanghai, China), vitamin C (A5960, Merck KGaA, Darmstadt, Germany), β-nicotinamide mononucleotide (NMN; HY-F0004, MedChemExpress, New Jersey, USA), and spermidine (S107071, Aladdin, Shanghai, China). Buffers and cell culture reagents included phosphate-buffered saline (PBS; G4202, Servicebio, Wuhan, China), Dulbecco's modified Eagle's medium (DMEM; MA0212, MeilunBio, Liaoning, China), and fetal bovine serum (FBS; F101-01, Vazyme, Nanjing, China). Cell viability was assessed using a Cell Counting Kit-8 (CCK-8; A311-02, Vazyme, Nanjing, China).Reagents used for quantitative real-time polymerase chain reaction (qPCR) included RNAiso Plus (9109, Takara Bio, Kusatsu, Japan), a reverse transcription kit (R323-01, Vazyme, Nanjing, China), and ChamQ Universal SYBR qPCR Master Mix (Q711-02-AA, Vazyme, Nanjing, China). Reagents used for Western blotting included RIPA lysis buffer (PS0013, Leagene, Beijing, China) and a bicinchoninic acid protein assay kit (BCA; E112-02, Vazyme, Nanjing, China). Primary antibodies against Oct-4 (bs-3816R, Bioss), Nanog (bs-10414R, Bioss), SOX-2 (bs-0523R, Bioss), CD63 (1:5000, 67605-1-Ig, proteintech, Wuhan, China), CD81(1:5000, 27855-1-AP, proteintech, Wuhan, China), TSG101(1:5000, 28283-1-AP, proteintech, Wuhan, China), Calnexin (1:10000, 10427-2-AP, proteintech, Wuhan, China) and β-tubulin (MA5-11732, Thermo Fisher Scientific, MA, USA) were used. Protein bands were visualized using an enhanced chemiluminescence reagent (ECL; MA0186-1, MeilunBio, Liaoning, China).Polycarbonate membranes used for the preparation of extruded nanovesicles had pore sizes and catalog numbers of 1 μm (610010), 0.8 μm (610009), 0.4 μm (610007), and 0.2 μm (610006), respectively, and were purchased from Avanti Polar Lipids (AL, USA). Dextran sulfate sodium (DSS; MB5535, MeilunBio, Liaoning, China) was used to establish the experimental animal model.Reagents used for flow cytometry included the Zombie Violet™ Fixable Viability Kit (423113, BioLegend, CA, USA), PE-conjugated anti-CD3 antibody (RRID: AB_2815096), APC-conjugated

anti-CD4 antibody (RRID: AB_2534398), FITC-conjugated anti-IL-17 antibody (RRID: AB_763581), APC-conjugated anti-FOXP3 antibody (RRID: AB_469457), and Foxp3/Transcription Factor Fixation/Permeabilization Buffer Set (00-5523-00, Thermo Fisher Scientific, MA, USA). Alcian blue staining solution (BB-44293, Bestbio, Shanghai, China) was used for histological staining.Reagents used for immunohistochemistry included bovine serum albumin (BSA; 4240GR200, BioFroxx), antibodies against Claudin-1 (GB12032, Servicebio, Wuhan, China), Occludin (GB151401, Servicebio, Wuhan, China), and ZO-1 (GB115686, Servicebio, Wuhan, China), as well as a 3,3′-diaminobenzidine chromogenic reagent kit (DAB; G1212, Servicebio, Wuhan, China).

Reagents used for agarose gel electrophoresis included YeaRed nucleic acid gel stain (10202ES76, Yeasen, Shanghai, China), 6× DNA loading buffer (D1010, Solarbio, Beijing, China), and a DNA ladder (D2000 Plus; M1070, Solarbio, Beijing, China). Ultrapure water was used throughout the experiments. Unless otherwise specified, all other chemicals were of analytical grade and used as received without further purification.

2.2 Apparatus and procedures

Sequential extrusion was performed using a lipid extruder (Hand Extruder-1 mL, Genizer LLC, California, USA) equipped with polycarbonate membranes of the pore sizes described above. Vesicles were purified using an ultracentrifuge (Optima XPN-90, Beckman Coulter, California, USA). Vesicle morphology and physicochemical properties were characterized by transmission electron microscopy (H-7650, Hitachi, Tokyo, Japan), nanoparticle tracking analysis (ZetaView, Particle Metrix, Germany), and zeta potential analysis (Zetasizer Nano, Malvern, UK), respectively.Agarose gels were visualized using a gel documentation system (Tanon 1600, Tanon, Shanghai, China). For 16S rRNA gene sequencing, paired-end sequencing with a read length of 250 bp (PE250) was performed on the Illumina NovaSeq platform. Sequencing data were processed using QIIME 2 with the DADA2 pipeline and/or VSEARCH, as appropriate.Fecal metabolomic analysis was performed using an ACQUITY ultra-

performance liquid chromatography system (ACQUITY UPLC, Waters, Milford, MA, USA) coupled to a high-resolution mass spectrometer (Orbitrap Exploris 120, BRE725531, Thermo Fisher Scientific, MA, USA). Raw metabolomic data were processed using Compound Discoverer 3.3 software (Compound Discoverer™ 3.3, version 3.3.2.31, Thermo Fisher Scientific, MA, USA).Statistical analyses were performed using GraphPad Prism. Image processing and densitometric quantification of Western blot bands were conducted using ImageJ.

2.3 Cell Culture and CCK-8 assay

MEF cells were cultured in DMEM supplemented with 10% FBS at 37°C in a 5% CO_2 incubator. Cells were seeded in 96-well plates at a density of 3,000 cells/well [1]. After 12 h of culture, cell growth status was observed. Cells were then grouped and treated with different drugs: VPA (1 mM, 0.5 mM, 0.25 mM, 0.1 mM), Vit C (1 mM, 100 μM, 10 μM, 1 μM), spermidine (100 μM, 10 μM, 1 μM, 100 nM), NMN (100 μM, 10 μM, 1 μM, 100 nM) [2-5]. After 72 h of drug intervention, CCK-8 reagent was added to each well, and incubation for another 1 h. Absorbance was measured at 450 nm using a microplate reader, and cell viability was calculated [6]. Cell viability (%) = (Absorbance of experimental group - Absorbance of blank group)/(Absorbance of control group - Absorbance of blank group) × 100% [6].

2.4 Isolation, purification, and identification of *L. rhamnosus*

First, *L. rhamnosus* indicated in commercial probiotic preparations were streaked onto MRS solid agar plates and incubated anaerobically at 37°C for 48 h [7]. Typical single colonies were picked and further purified by triple streak plating to obtain monoclonal strains [8]. Purified strain underwent preliminary Gram staining screening, and genomic DNA was extracted. After PCR amplification and 16S rRNA sequencing confirming the strain as *L. rhamnosus*, and then for further used. The identified strain was inoculated into MRS liquid medium and cultured anaerobically at 37 °C to the logarithmic growth phase. *L. rhamnosus* were collected by centrifugation, washed with

PBS, and re-suspended as needed *L. rhamnosus* suspension for oral gavage was adjusted to the target concentration by plate counting to determine colony-forming units (CFU). For long-term storage, bacterial suspensions were mixed with a cryoprotectant containing 50% glycerol and stored frozen at -80 °C [9].

2.5 Agarose gel electrophoresis

PCR products from bacterial cultures were detected by agarose gel electrophoresis, agarose was weighed and dissolved in 1 × TAE buffer to prepare 1% gel [10]. After complete dissolution by heating and cooling to about 60°C, YeaRed Nucleic Acid Gel Stain (10202ES76, Yeasen, Shanghai, China) was added, poured into a gel-casting tray with a comb, and solidified at room temperature. The gel was placed in an electrophoresis chamber with 1 × running buffer. Samples were mixed with 6 × DNA loading buffer (D1010, Solarbio, Beijing, China) and loaded, along with DNA Ladder (D2000 plus, M1070, Solarbio, Beijing, China) as the molecular weight standard[11]. Electrophoresis was performed at 100 V for 30 min [12]. Bands were visualized and imaged using a gel imaging system (Tanon 1600, Tanon, Shanghai, China) to determine target fragment size and specificity.

2.6 Primary MEF cell isolation

Pregnant C57BL/6 dams at gestational day 13 were euthanized according to animal ethics requirements. Uterine horns were isolated aseptically, and embryos were removed and placed in pre-cooled PBS. After removing embryonic membranes and placenta, the embryos' heads, limbs, and internal organs were excised (to reduce neural and blood cell contamination) [13]. The remaining trunk tissue was thoroughly rinsed with PBS and minced into approximately 1 mm^3 pieces. 0.25% Trypsin-EDTA was added, and digestion proceeded at 37 °C for 15 min with gentle pipetting to aid dissociation [13, 14]. Digestion was stopped by adding DMEM containing 10 % FBS. To increase cell yield, digestion could be repeated 1-2 times. Digestates were combined, filtered through a 70 μm cell strainer, and centrifuged at 400 g for 5 min to collect the cell pellet [15]. Cells were resuspended in complete medium and seeded into culture flasks, incubated

at 37 °C, 5% CO_2. After 24 h, the medium was changed to remove non-adherent cells and debris. When cells reached 80-90% confluence, they were passaged using trypsin. MEF cells from passages 2-4 (P2-P4) were used for subsequent studies.

2.7 Immunofluorescence staining

Mouse colon paraffin sections were deparaffinized in xylene and rehydrated through graded ethanol, followed by heat-induced antigen retrieval. After cooling and washing with PBS, tissue was permeabilized using Triton X-100 and blocked with 5% BSA at room temperature for 60 min [16, 17].

After removing the blocking solution, sections were incubated overnight at 4°C in the dark with a mixture of corresponding primary antibodies. 24 hour later, sections were thoroughly washed with PBS containing 0.05% Tween-20, followed by incubation with appropriate fluorescently labeled secondary antibodies at room temperature in the dark for 1 h. Nuclei were counterstained with DAPI for 10 min [16, 17]. Finally, sections were mounted with an anti-fade mounting medium. Images were captured using a fluorescence microscope with consistent acquisition parameters.

Results

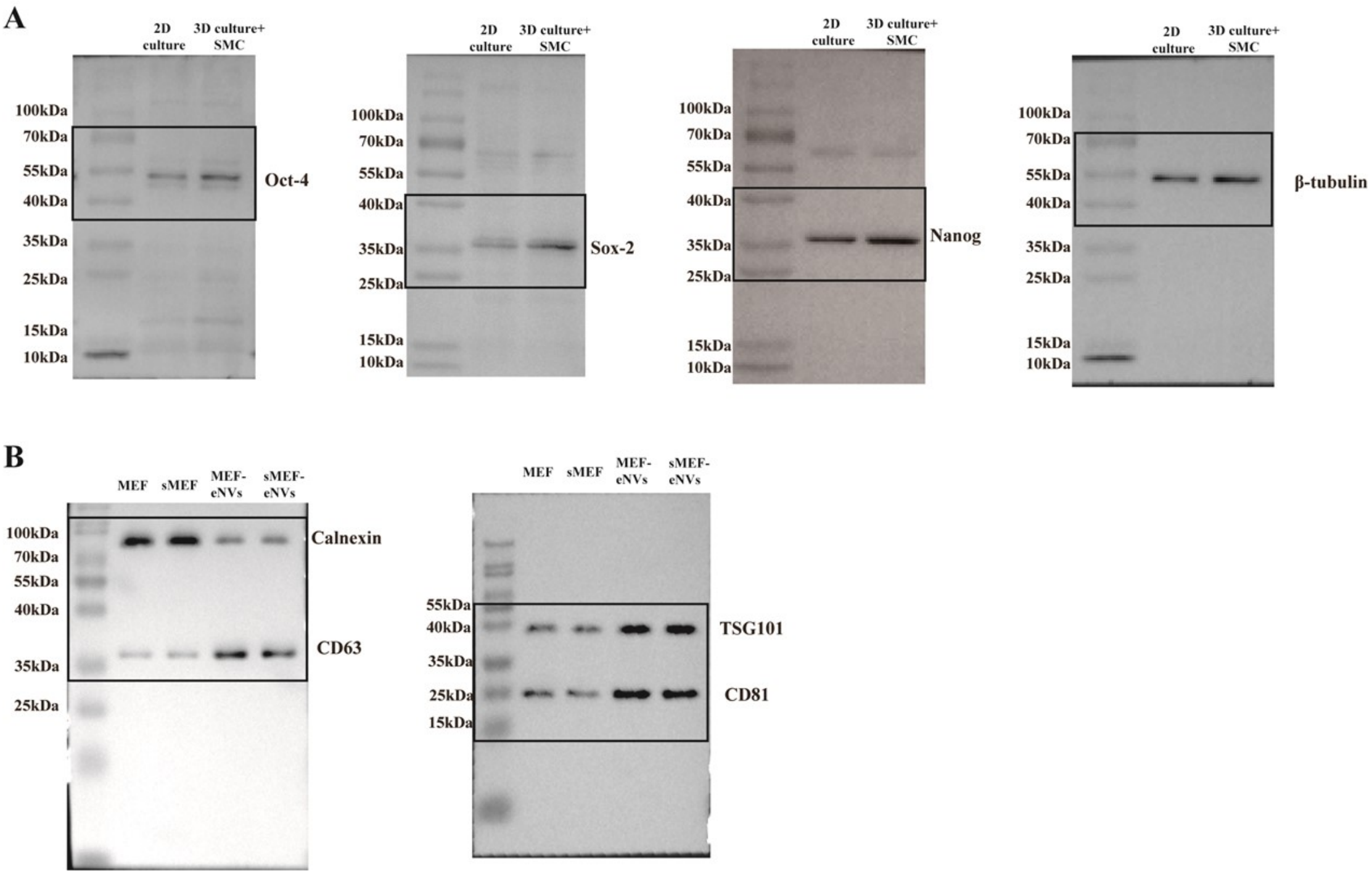

Figure S1. (A) Representative uncropped and unprocessed western blot analysis of Oct-4, Nanog and Sox-2 in MEFs cultured under conventional 2D condition or in 3D culture corresponding to Figure 1D. (B) Representative uncropped and unprocessed western blot analysis of EV-associated markers CD63, CD81, TSG101, and negative markers Calnexin corresponding to Figure 2D.

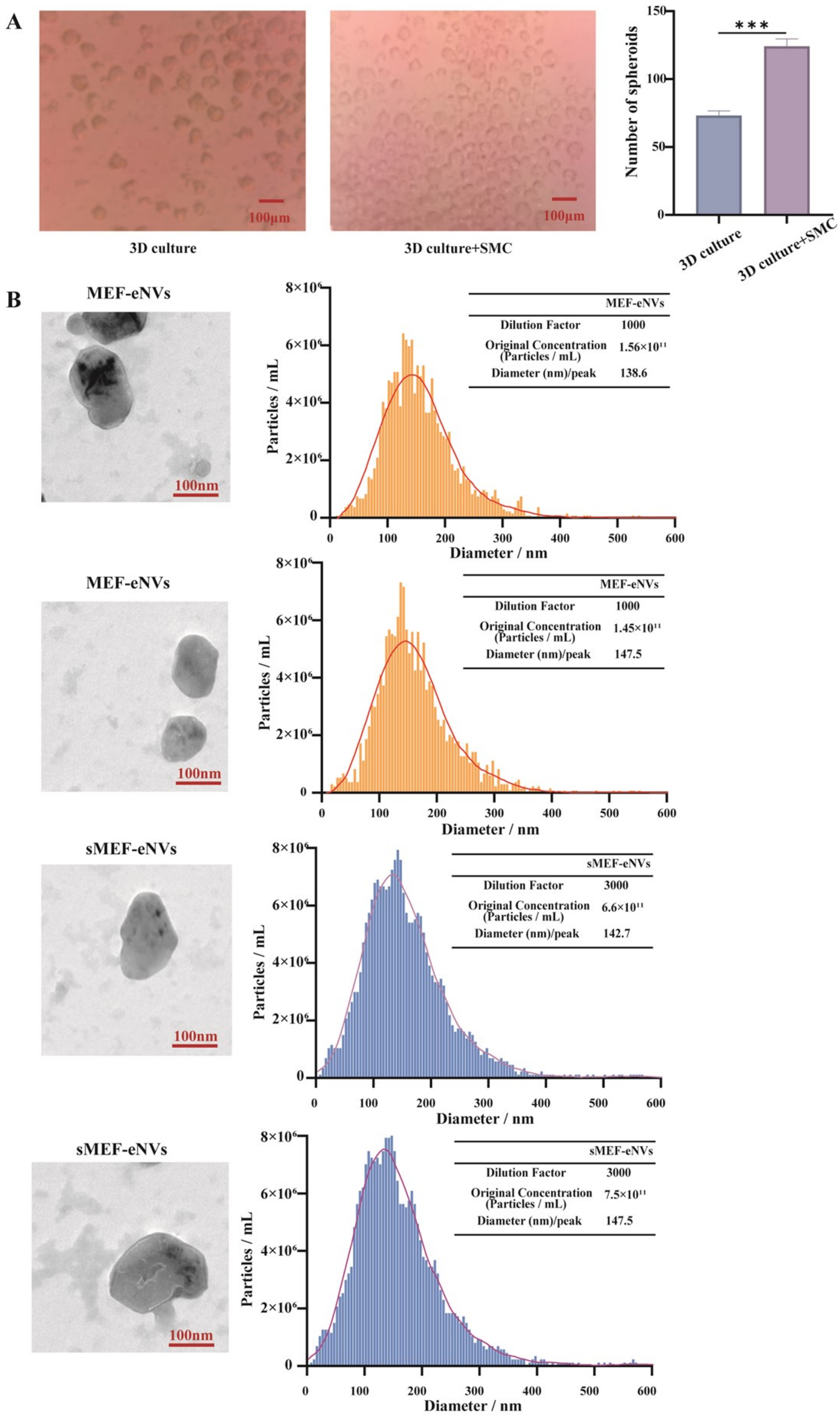

Figure S2. (A)Representative bright-field images of MEF spheroids formed in 3D culture with or without SMC, and quantification of spheroid number. (B) Additional batch reproducibility of MEF-eNVs and sMEF-eNVs' characterization.

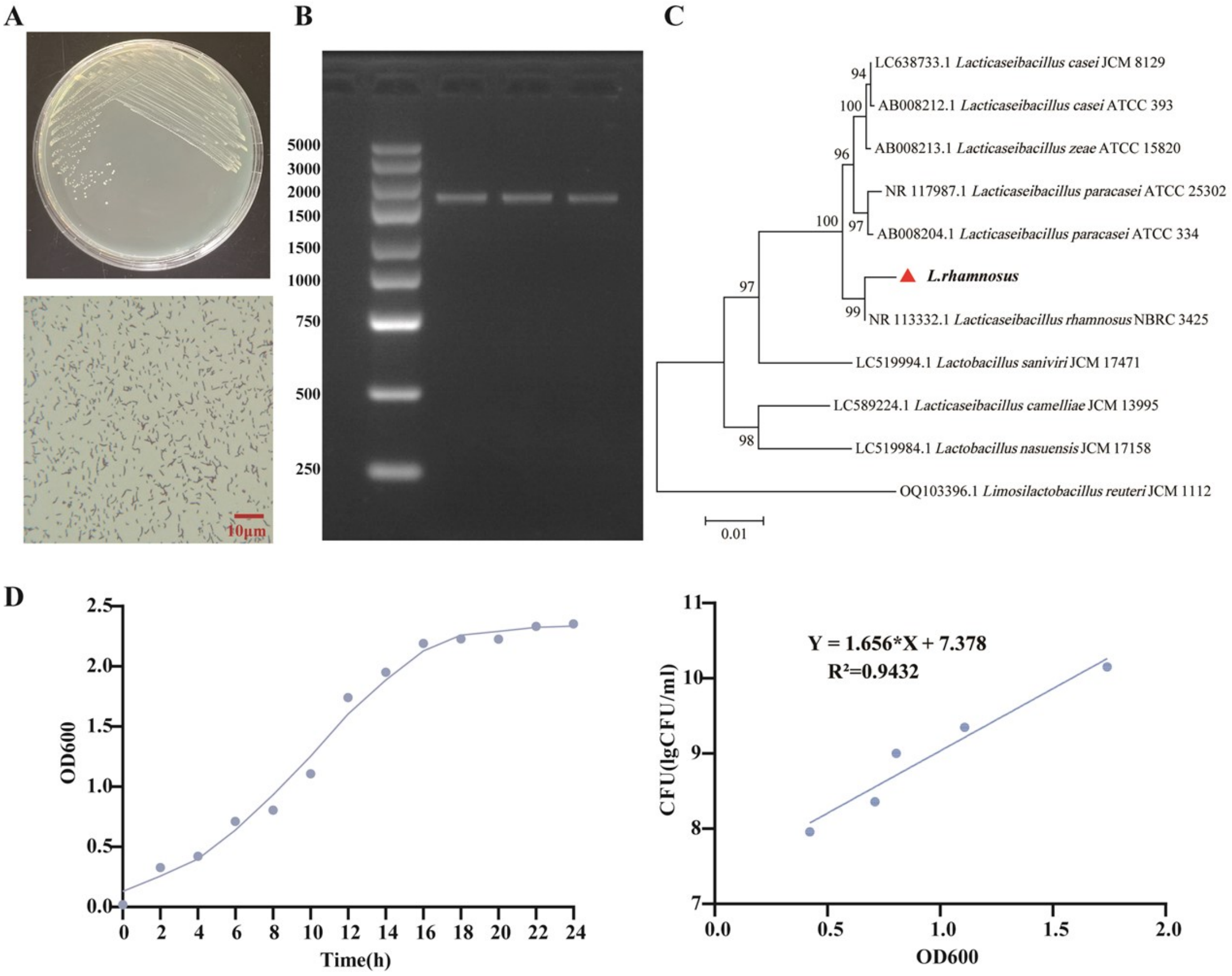

Figure S3. Isolation, identification, and growth characteristics of *L. rhamnosus.* (A) Colony morphology on solid medium (top) and microscopic morphology of bacteria (bottom). (B) Agarose gel electrophoresis of PCR amplicons (molecular size marker shown on the left). (C) 16S rRNA gene-based phylogenetic tree, the isolate is indicated by a red triangle. Bootstrap values are shown at branch nodes, scale bar indicates substitutions per site. (D) Growth curve monitored by OD_{600} over time and linear regression between OD_{600} and viable counts (CFU/mL).

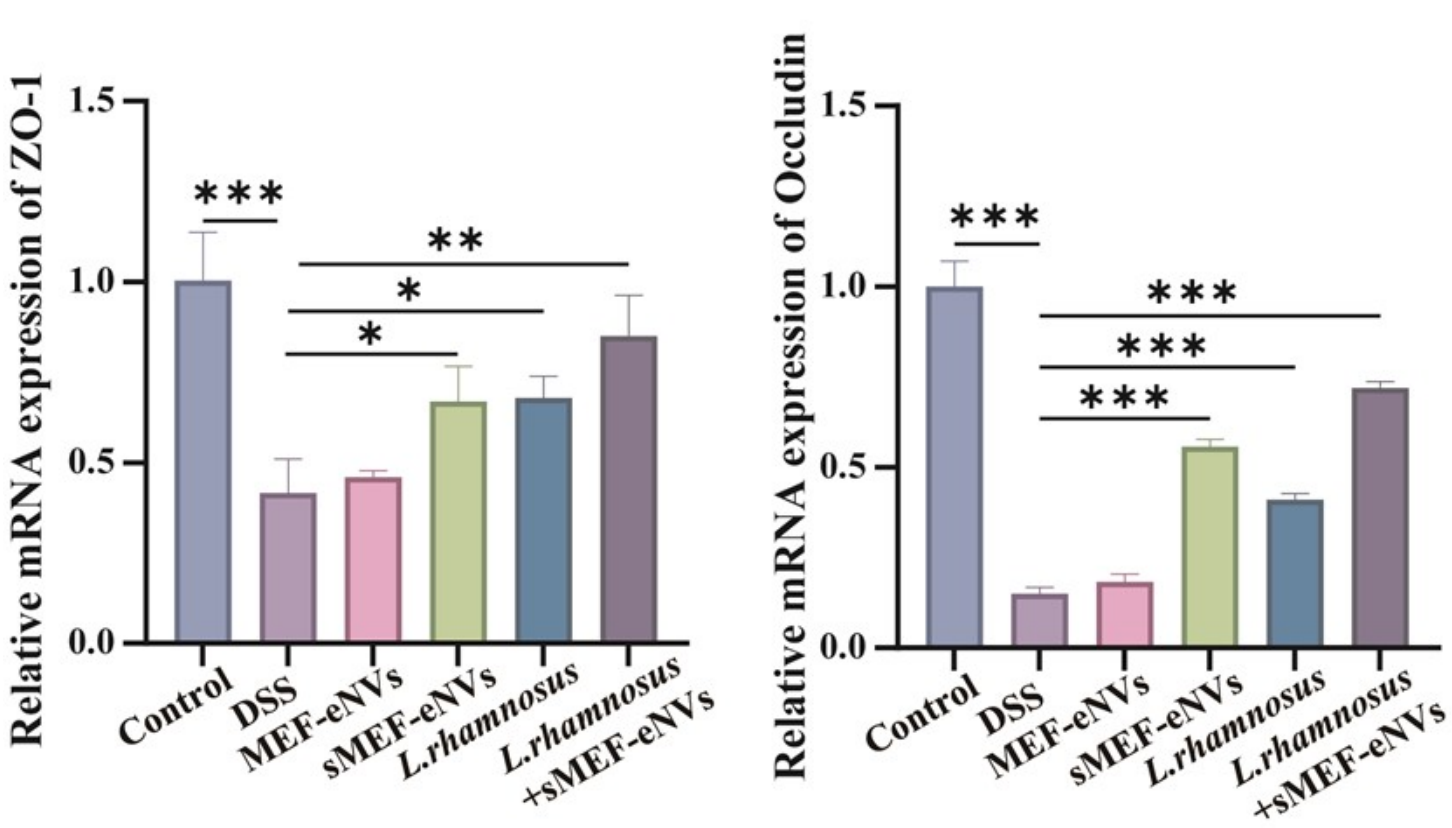

Figure S4. qRT-PCR quantification of related mRNA expression. Bars represent mean with error bars as shown. Data are presented as mean ± SD from n = 3 independent experiments. Data was analyzed using one-way analysis of variance. *ns*, not significant; **P* < 0.05, ***P* < 0.01, ****P* < 0.001.

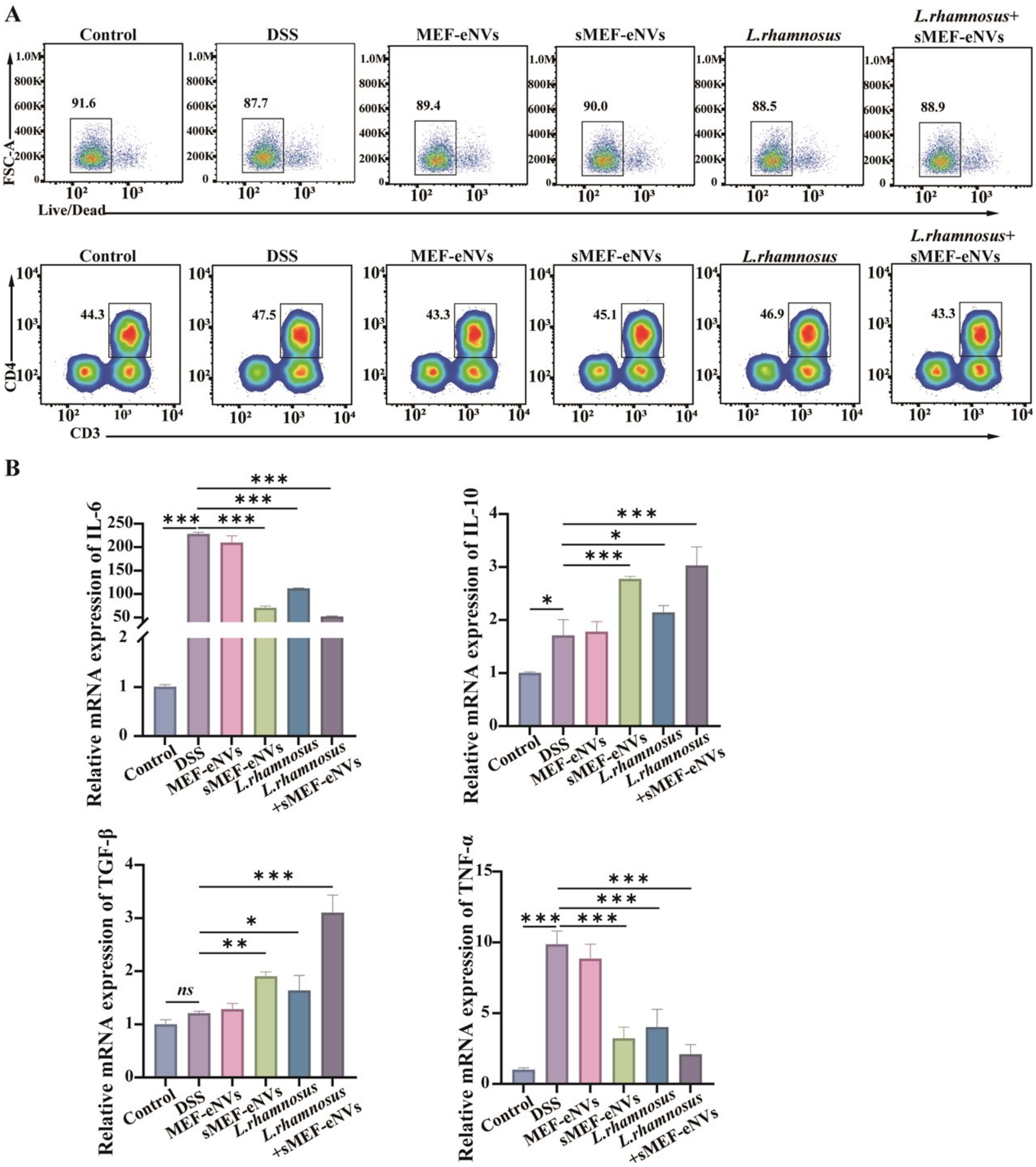

Figure S5. (A) Representative gating strategy and plots: live cells, $CD3^{+}CD4^{+}$ T cells. (B) qRT-PCR analysis of cytokine-related genes in colon tissue, including IL-6, IL-10, TGF-β, and TNF-α. Bars represent mean with error bars as shown. Data are presented as mean ± SD from n = 3 independent experiments. Data was analyzed using one-way analysis of variance. *ns*, not significant; **P* < 0.05, ***P* < 0.01, ****P* < 0.001.

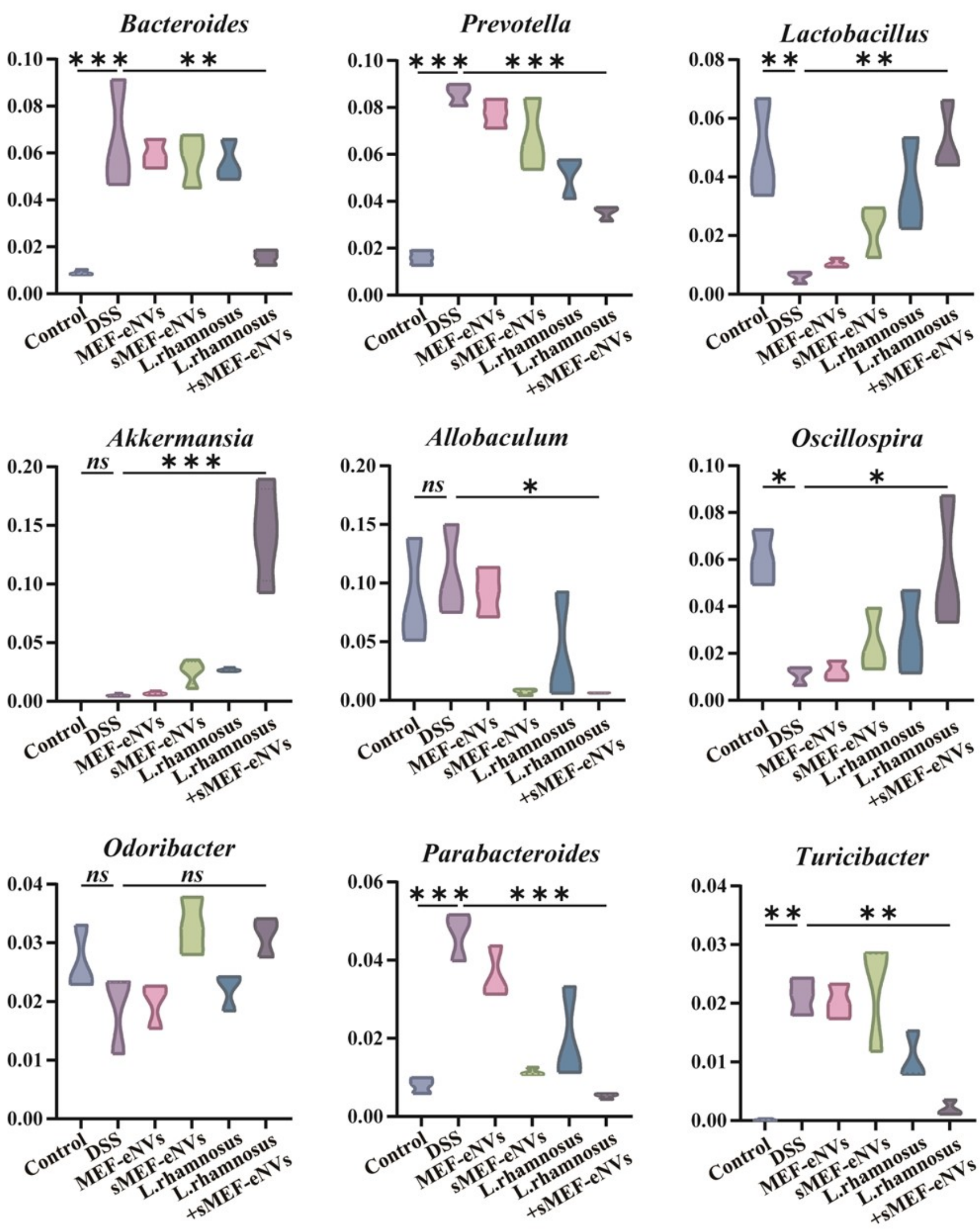

Figure S6. Differential relative abundance of representative genera. n = 4 mice per group. Data was analyzed using one-way analysis of variance. *ns*, not significant, *P < 0.05, **P < 0.01, ***P < 0.001.

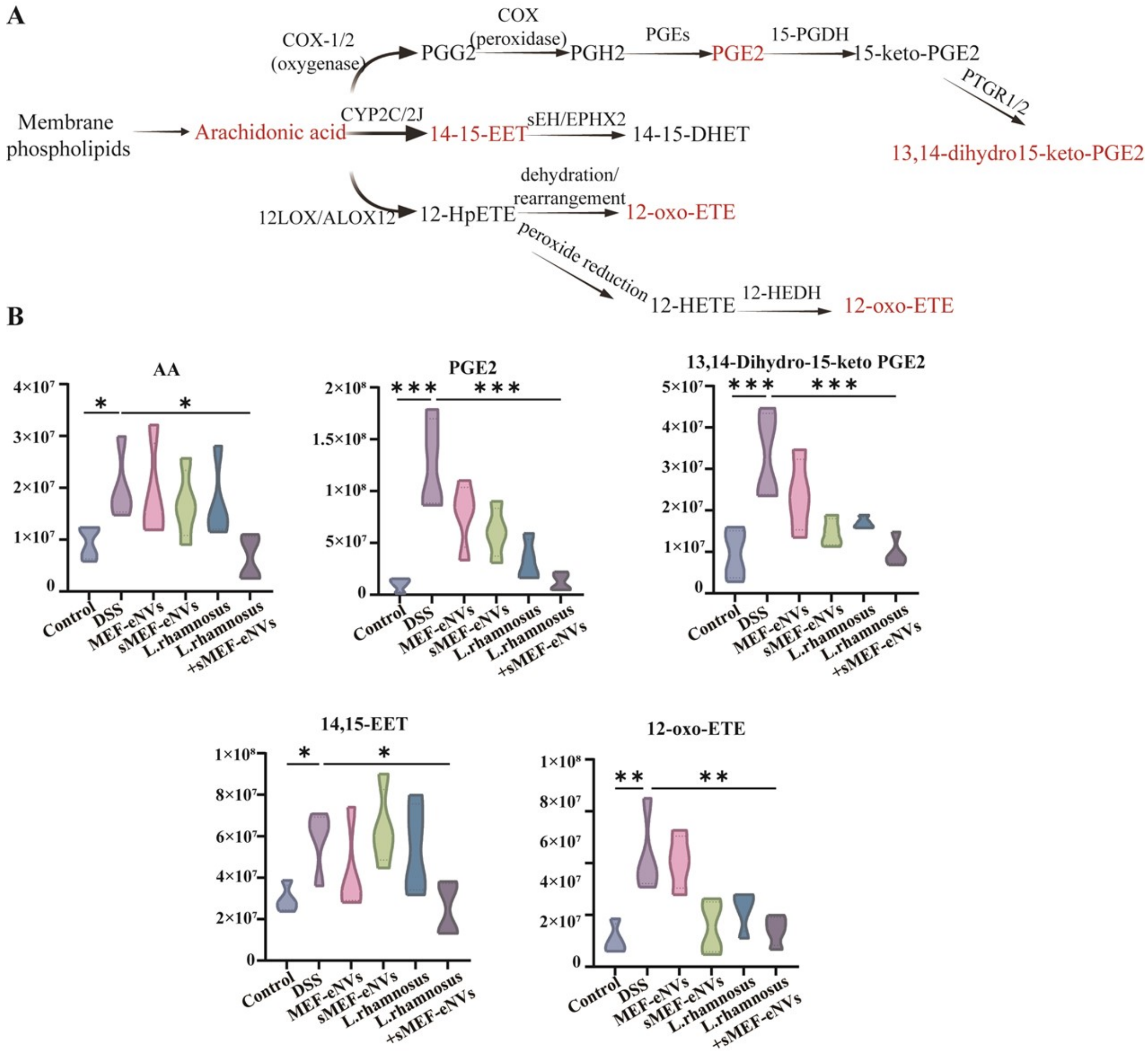

Figure S7. (A) Schematic overview of arachidonic acid metabolic routes, including COX-derived prostaglandin metabolism and CYP-derived epoxide pathways.(B) Violin plots showing relative levels of representative metabolites (arachidonic acid, PGE2, 13,14-dihydro-15-keto-PGE2, 12-oxo-ETE, and 14,15-EET) across groups. n = 4 mice per group. Data was analyzed using one-way analysis of variance. *ns*, not significant, *$P < 0.05$, **$P < 0.01$, ***$P < 0.001$.

References

1. L. Gong, D. Cui, D. Liu, X. Shen, H. Pan, X. Xiong and Y. Zhao, Cell Death Dis., 2022, 13, 788.
2. M. Li, Z. Qiao, J. Li, H. Zhou, D. Huang, Y. Cai, X. Li, Z. Zhang, J. Zhou and J. Zhou, Transl. Psychiatry, 2025, 15, 271.
3. A. Prasad, D. Rathi, M. Sedlářová, R. R. Manoharan, E. Průdková and P. Pospíšil, Biochem. Biophys. Rep., 2024, 37, 101622.
4. D. Lumkwana, C. Peddie, J. Kriel, L. L. Michie, N. Heathcote, L. Collinson, C. Kinnear and B. Loos, Front. Cell Dev. Biol., 2022, Volume 10 - 2022.
5. Z. Zheng, X. Wang, L. Ouyang, W. Chen, L. Zhang and Y. Cao, Antioxidants (Basel), 2023, 12.
6. Y. Huang, J. Bai, B. Ran, J. Li, B. Chen, Z. Chen, J. Chen, Y. Wang, J. Li, Q. Dong, Q. Wei, D. Cao and L. Liu, Front. Pharmacol., 2025, Volume 16 - 2025.
7. A. Navarré, T. Nazareth, C. Luz, G. Meca and L. Escrivá, Food Funct., 2024, 15, 8087-8103.
8. R. Srivastava, B. Chamadia, P. V. Behare, K. Chauhan, S. Kumar, G. Mondal and N. Tyagi, AMB Express, 2025, 15, 140.
9. R. D. Au - Ayivi, A. Au - Edwards, D. Au - Carrington, A. Au - Brock, A. Au - Krastanov, A. S. Au - Eddin and S. A. Au - Ibrahim, JoVE, 2022, https://doi.org/doi:10.3791/63314e63314.
10. Q. Chen, Z. Fang, Z. Yang, X. Xv, M. Yang, H. Hou, Z. Li, Y. Chen and A. Gong, J. Agric. Food Chem., 2024, 72, 14713-14726.
11. X. Guo, Y. Huang, J. Wang, S. Fu, C. Wang, M. Wang, C. Zhou, X. Hu, T. Wang, W. Yang and F. Han, Front. Plant Sci., 2023, Volume 14 - 2023.
12. H. Chen, Y. Sheng, S. Wang, Y. Chen, Z. Qiao, H. Guo and H. Dong, Front. Microbiol., 2025, Volume 16 - 2025.
13. S. H. Pan, N. Zhao, X. Feng, Y. Jie and Z. B. Jin, Sci. Adv., 2021, 7.
14. M. R. O'Dwyer, M. Azagury, K. Furlong, A. Alsheikh, E. Hall-Ponsele, H. Pinto, D. V. Fyodorov, M. Jaber, E. Papachristoforou, H. Benchetrit, J. Ashmore, K. Makedonski, M. Rahamim, M. Hanzevacki, H. Yassen, S. Skoda, A. Levy, S. M. Pollard, A. I. Skoultchi, Y. Buganim and A. Soufi, Nature, 2025, 638, 251-260.
15. A. Morita, M. Ishii, T. Asakura, M. Yotsukura, A. E. Hegab, T. Kusumoto, H. Namkoong, T. Ogawa, Y. Nakatake, M. Oda, F. Saito, H. Kamata, J. Hamamoto, S. Okamori, T. Ebisudani, H. Yasuda, S. Sugimoto, Y. Kuze, M. Seki, Y. Suzuki, N. Hasegawa, H. Asamura, H. Watanabe, M. Ko, T. Sato, M. Ieda and K. Fukunaga, npj Regener. Med., 2025, 10, 30.
16. K. Kim, J. H. An, S. M. Park, G. Lim, K. W. Seo and H. Y. Youn, J Vet Sci, 2023, 24, e52.
17. D. Dikovskaya, R. Pemberton, M. Taylor, A. Tasegian, P. Bhattacharya, K. Zeneviciute, E. M. Sammler, A. J. M. Howden, D. R. Alessi and M. Swamy, EMBO Rep., 2025, 26, 3327-3356.
